# Single-nucleus RNA sequencing reveals distinct pathophysiological trophoblast signatures in spontaneous preterm birth subtypes

**DOI:** 10.1186/s13578-024-01343-0

**Published:** 2025-01-07

**Authors:** Cherilyn Uhm, Jianlei Gu, Weina Ju, Stephanie Pizzella, Hande Oktay, Joyce Yao-Chun Peng, Sararose Guariglia, Yong Liu, Hongyu Zhao, Yong Wang, Ramkumar Menon, Nanbert Zhong

**Affiliations:** 1 New York State Institute for Basic Research in Developmental Disabilities, Staten Island, NY 10314 USA; 2 School of Public Health, Yale University, New Haven, CT 06520-0834 USA; 3 Department of Obstetrics and Gynecology, School of Medicine, Washington University, St. Louis, MO 63110-1010 USA; 4 Singulomics Corporation, Bronx, NY 10461 USA; 5 The University of Texas Medical Branch at Galveston, Galveston, TX 77555-0144 USA

## Abstract

Spontaneous preterm birth (sPTB) poses significant challenges, affecting neonatal health and neurodevelopmental outcomes worldwide. The specific effects of placental trophoblasts on the pathological development of sPTB subtypes—preterm premature rupture of fetal membranes (pPROM) and spontaneous preterm labor (sPTL)—are not fully understood, making it crucial to uncover these impacts for the development of effective therapeutic strategies. Using single-nucleus RNA sequencing, we investigated transcriptomic and cellular differences at the maternal–fetal interface in pPROM and sPTL placentas. Our findings revealed distinct trophoblast compositions with pPROM characterized predominantly by extravillous trophoblasts (EVTs), while sPTL showed an abundance of syncytiotrophoblasts (STBs). Through cell differentiation and cell-to-cell communication analyses, other distinguishing factors were also found. In pPROM, heightened inflammation, oxidative stress, and vascular dysregulation with key pathways including tumor necrosis factor signaling, matrix metalloproteinase activation, and integrin-mediated cell adhesion, highlighted an invasive EVT profile potentially driven by hypoxic conditions and immune cell recruitment. In contrast, sPTL was marked by increased smooth muscle contraction, vascular remodeling, and altered signaling dynamics involving fibroblasts, including TGF-β and WNT pathways. Our study highlights the critical need to distinguish sPTB subtypes to improve diagnostic precision and therapeutic targeting. The molecular insights gained provide a foundation for future investigations aimed at functional validation of key pathways and exploration of trophoblasts on the development of sPTB. Ultimately, these findings pave the way for more personalized and effective interventions to mitigate adverse outcomes associated with preterm birth.

**Supplementary Information:**

The online version contains supplementary material available at 10.1186/s13578-024-01343-0.

## Background

Spontaneous preterm birth (sPTB) is a complex and widespread syndrome that significantly contributes to neonatal morbidity and mortality worldwide [1–3]. In 2020 alone, ~ 13.4 million infants were born before 37-week gestation [4]. Additionally, in 2019, around 900,000 children under the age of five years died from complications related to preterm birth, including lower respiratory infections, intrapartum-related events, and other perinatal causes [5]. Beyond the immediate postnatal risks to infant survival, sPTB poses lasting challenges. It can disrupt the intricate coordination required in the final weeks of gestation, potentially interfering with the established sequence of fetal brain development and affecting multiple aspects of a child’s development [6]. Among these, disruptions in the highly orchestrated and time-sensitive process of fetal brain development are associated with an increased risk of neurodevelopment disorders, learning difficulties, and behavioral and social challenges [7].

Considering the clinical impact of sPTB, it is essential to distinguish between its two main subtypes: preterm premature rupture of membranes (pPROM) and spontaneous preterm labor (sPTL). Many studies have failed to differentiate between the sPTB subtypes, preterm premature rupture of fetal membranes (pPROM), and spontaneous preterm labor (sPTL), the latter being characterized by muscle contractions rather than a membrane rupture [8–11]. sPTB is a clinical syndrome with various causes [12], in which both pPROM and sPTL are complex conditions. Clinically, pPROM may be associated with infection and inflammation, resulting from the activation of cytokines, matrix metalloproteinases, and apoptotic pathways [13]. sPTL, on the other hand, may arise from multiple factors that stimulate cervical ripening and uterine contractility [14, 15].

Infection and/or inflammation are the most common etiological factors that may result in pPROM and sPTL at an early stage of pregnancy, and these could have a pathological impact on intrauterine fetal neurodevelopment. However, the pathogenic mechanisms and links to neuronal development within the maternal–fetal interface in sPTB are not fully understood, partly due to the lack of powerful and accurate methods for real-time intrauterine assessment of fetal neurodevelopment during pregnancy.

Studies using genetic, genomic, epigenetic, and transcriptomic approaches in sPTB have identified several genetic loci potentially involved in premature parturition. However, the findings regarding the maternal–fetal interface have been inconsistent and lack functional validation [16, 17]. Much of the research on sPTB has focused on correlating monogenic factors, such as single-omic data generated from genome-wide variation in pregnant women. These studies have also highlighted the crucial role of cellular components within the maternal–fetal interface, particularly the trophoblast populations [18–20]. The key trophoblast cell types, cytotrophoblast (CTB), syncytiotrophoblast (STB), and extravillous trophoblast (EVT) play distinct but interconnected roles in placental function and fetal development [21, 22]. The CTB and the CTB within the villous layer (VCT) serve as the progenitor population, differentiating into STB, which is responsible for maintaining the nutrient and gas exchange necessary for pregnancy maintenance. EVT differentiation occurs at the villi, with EVTs invading the maternal decidua and remodeling spiral arteries, thereby providing blood supply to the fetus.

Understanding these intricate processes is essential for unraveling the pathological mechanisms behind sPTB. By considering the distinct clinical subtypes of sPTB, we aimed to provide a more nuanced understanding of the molecular and pathological differences that underlie sPTB and its diagnostic markers with a single-cell transcriptomic approach. For which, the single-cell RNA sequencing (scRNA-seq) offers a unique opportunity to explore cell-type-specific transcriptomic landscapes within the maternal–fetal interface [23].

## Materials and methods

### Study design

The aim of this study was to conduct a comprehensive analysis of the pathophysiology underlying the subtypes of sPTB, specifically focusing on pPROM and sPTL, and provide a new avenue for exploration. We utilized snRNA-seq to examine differential gene expression and other molecular differences between the sPTB subtypes.

### Sample collection

Human fetal membrane samples were obtained from the University of Texas Medical Branch at Galveston. Initially, nuclei from nine frozen human fetal membrane samples were isolated and processed for sequencing. Due to quality concerns, one pPROM sample was excluded due to lower data quality, and one sPTL sample was removed for having a lower uniparental mitochondrial inheritance count compared to other samples (Table 1).

**Table 1:** Sample number, maternal age, history of pregnancy (Hx pregnancy), and gestational age of control, pPROM, and sPTL pregnancy groups

Group	Sample number	Maternal age	Hx pregnancy	Gestational age
Control	1	39	G6P4	39.0
	2	23	G3P2	39.0
	3	29	G4P2	39.0
pPROM	1	30	G4P2	34.3
	2	24	G3P2	33.3
sPTL	1	23	G1P0	40.4
	2	34	G3P1	40.2

### Single-nucleus RNA sequencing

In an earlier study, we applied scRNA-seq to examine freshly collected chorionic villi samples for spontaneous miscarriages [23]. In this study, we employed single-nucleus RNA sequencing (snRNA-seq) [24] and studied pre-banked human placentas [25]. Instead of isolating cells, nuclei used for isolation of RNAs were isolated from freshly frozen placental tissues and subjected to library construction and RNA sequencing for snRNA-seq. Briefly, the placental tissue samples were homogenized and lysed with Triton X-100 in RNase-free water for nuclei isolation. The isolated nuclei were purified, centrifuged, and resuspended in PBS with BSA and RNAse inhibitor. Nuclei were diluted to 700 nuclei/µl and loaded into the 10 × Genomics Chromium Controller to encapsulate single nuclei into droplet emulsions, following the manufacturer’s recommendations (10 × Genomics, Pleasanton, CA, USA). Raw sequencing data for the single-nucleus transcriptome was converted into FASTQ format and processed through the Cell Ranger pipeline (10X Genomics Cell Ranger 7.1.0) to obtain barcodes, features, and matrix files, which were subsequently imported into R for analysis. The datasets supporting the conclusions of this article are available in the NCBI Gene Expression Omnibus under accession number GSE174399 (https://www.ncbi.nlm.nih.gov/), as well as provided in the Appendices (Data file S1–S4).

Data processing, including quality control and preprocessing, followed previously established protocols [26]. Cells were excluded if they expressed fewer than 200 genes, had fewer than 1000 total UMI counts, or exhibited greater than 10% total mitochondrial gene expression. Additional steps included dataset merging, normalization, variable features identification, scaling, principal component analysis, neighborhood and cluster identification, and Uniform Manifold Approximation and Projection (UMAP), performed using the Seurat v4 R package (https://satijalab.org/seurat/). Prior to dataset integration, the marker genes for each cluster were identified using the ‘FindAllMarkers’ function within ‘Seurat.’ Cluster annotation was performed by matching clusters to known cell types by using a reference placenta gene panel, as we described [23] (Data file S1–S3). To maintain consistency across conditions, we retained the original ‘Seurat’ objects for individual conditions, then merged them for downstream processing to explore batch effects and ensure uniformity. The same annotation approach was applied to the merged dataset to categorize clusters (Data file S4).

Kyoto Encyclopedia of Genes and Genomes (KEGG) analysis: To gain insight into the biological pathways associated with the different conditions, KEGG pathway enrichment analysis (http://www.kegg.jp/kegg) was conducted on the individual ‘Seurat’ datasets. We began by converting the top 10 differentially expressed genes (DEGs) (p < 0.01) in each cluster, ranked by highest to lowest average log2-fold change, as identified using the ‘FindAllMarkers’ function in ‘Seurat,’ to EntrezIDs. These were mapped to the human genome-wide annotation using the ‘org.Hs.eg.db’ package. The ‘clusterProfiler’ package was used to identify the top 20 KEGG pathways (p < 0.05), including details on gene ratio, gene count, and p-values, using the ‘enrichKEGG’ function.

Trajectory and pseudotime ordering: We modeled cellular trajectories and inferred pseudotime by using the ‘Monocle 3’ R package (https://cole-trapnell-lab.github.io/monocle3/). The individual ‘Seurat’ objects were converted to ‘CellDataSet’ objects and root cells, or starting points were selected through ‘Monocle 3’ to order cell types along a developmental trajectory. The ‘graph_test’ function was employed to identify genes that significantly changed along the trajectory, which were further analyzed based on q-values.

Cell-to-cell communication analysis: Cell-to-cell communication was assessed using the ‘CellChat’ R package (http://www.cellchat.org/). The merged dataset was divided into the condition-specific subsets and converted into ‘CellChat’ objects, grouped by the cell type annotations from the original merged dataset. Further processing involved setting the ligand-receptor interaction database to ‘CellChatDB.human,’ identifying over-expressed genes and interactions, computing communication probability, and aggregating networks. Cophenetic and silhouette indexes were based on the non-negative matrix factorization (NMF) R package [27]. Additional visualization models within ‘CellChat,’ such as scatterplots comparing the outgoing and incoming interaction strengths, heatmaps indicating outgoing and incoming signaling pathways, and bubble plots highlighting the communication probabilities, aided in the interpretation and comparative analysis of cell-to-cell interactions.

## Results

### Variation of cell subtypes and differential gene expression: pPROM *vs*. sPTL

**Fig. 1:**
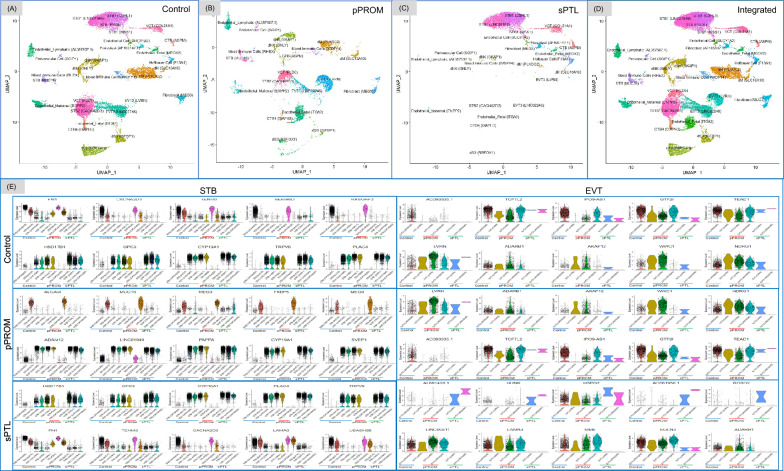
Cell clusters characterized by snRNA-seq among sPTB placentas. UMAPs of cell clusters were annotated by snRNA-seq among groups of the control (**A**), premature preterm rupture of membranes (pPROM) (**B**), spontaneous preterm labor (sPTL) (**C**), and integrated A, B, and C conditions (**D**). Violin plots present gene expressions of the top five scored upregulated or downregulated genes for all cell clusters of syncytiotrophoblasts (STBs) and extravillous trophoblasts (EVTs). The upregulated genes are lined up in upper rows, and the downregulated genes are in lower rows in each group (**E**)

A broad range of differences in cell subtypes, as characterized by snRNA-seq, was observed between pPROM and sPTL. UMAP showcased diverse cell types, including trophoblast cells: CTBs, VCTs, STBs, and EVTs; immune cells: decidual macrophages (dMs), decidual natural killer (dNK), Hofbauer, and mixed immune; endothelial cells: fetal, maternal, and lymphatic; as well as fibroblasts and perivascular (PV) cells (Fig. 1A–D). In a previous study, we identified three distinct types of EVT and two types of STB [23], but in this study, we observed different gene expression patterns of the identical cell type, such as EVT3 consisting of two unique clusters.

When the UMAP findings were split by the pPROM and sPTL conditions, we noticed key distributional differences between the preterm conditions compared to full-term control (Fig. 1A–C). While pPROM included various immune cells, such as dNK, dM, and mixed immune cells, these clusters exhibited different gene expression patterns compared to the Hofbauer cell cluster observed in sPTL, suggesting that immune cells infiltrating the pPROM placental environment may be replacing or masking the normally expected Hofbauer cells. Furthermore, there were notable distributional differences between pPROM and sPTL. Despite limited overlap, a clear pattern emerged in the EVT and STB cell types. In the pPROM condition, EVTs were prevalent, whereas most STB cell types were absent. In contrast, sPTL exhibited an abundance of various STB cell subtypes but lacked EVT cells. This difference is unlikely related to gestational age, as all samples were taken from pregnancies at approximately the same gestational week. Differential gene expression in STB and EVT clusters across the three groups further supports the observed differences, highlighting the presence of EVT clusters in pPROM and of STB clusters in sPTL (Fig. 1E). (For enlarged versions of all figures throughout the results, please refer to Supplementary Figures.)

### Gene dynamics in sPTB through pathway and trajectory analysis

**Fig. 2:**
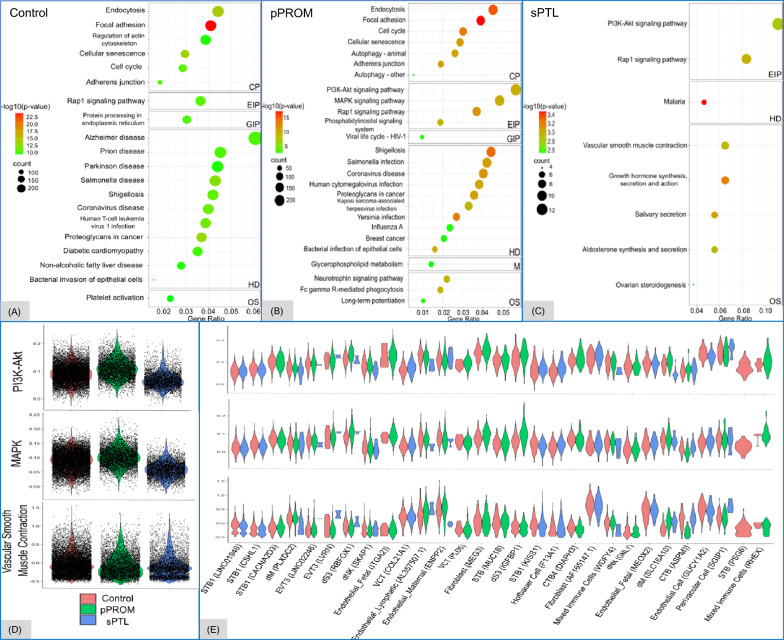
Pathophysiological pathways. Pathophysiological pathways, which were identified to be statistically significant (adjusted p < 0.05, FDR correction) in the premature preterm rupture of membranes (pPROM) and spontaneous preterm labor (sPTL) groups, when compared to the control group, are shown with bubble plots. The horizontal axis represents the gene ratio, and the vertical axis represents the enriched pathways. The color scale shows the − log10(p-value), and the size of the bubble indicates the gene count for each pathway. The labels on the right of each bubble chart represent the KEGG subcategories: CP = cellular processes, EIP = environmental information processing, HD = human diseases, GIP = genetic information processing, and OS = organismal systems (**A–C**). Violin plots present expression levels for genes significantly associated with a uniquely enriched KEGG pathway, either split by clinical features (**D**) or by cell clusters (**E**). The phosphatidylinositol 3-kinase/ protein kinase B (PI3K-Akt) pathway is associated with pPROM and sPTL; the mitogen-activated protein kinase (MAPK) pathway, with pPROM; and the vascular smooth muscle contraction pathway, with sPTL

Functional differences between pPROM and sPTL were explored using KEGG pathway enrichment and trajectory analysis [28, 29]. The phosphatidylinositol 3-kinase/protein kinase B pathway, which is crucial for maternal metabolism, placental-fetal growth, morphology, and nutrient transport, was enriched in both pPROM and sPTL conditions. This pathway displayed consistent enrichment within PV (*SGIP1*), fibroblast (*MEG3*), endothelial_fetal (*ITGA2*), and endothelial (*GUCY1A2*) cells. The mitogen-activated protein kinase pathway, which plays a role in cell proliferation, differentiation, and apoptosis, was exclusively enriched in pPROM, particularly in dS3, EVT3 (*LVRN*), and mixed immune (*RHEX*) cell clusters. In contrast, the vascular smooth muscle contraction pathway was uniquely enriched in sPTL, with high expression in fibroblast (*AF165147.1*), endothelial_fetal (*MEOX2*), endothelial (*GUCY1A2*), EVT3 (*LVRN*), CTB (*ASPM*), and PV (*SGIP1*) cells (Fig. 2).

**Fig. 3:**
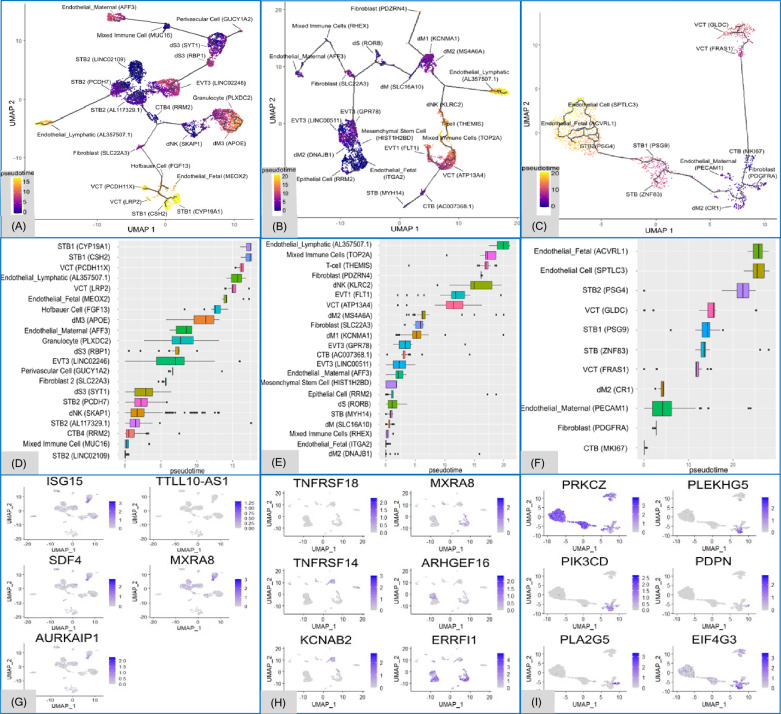
Trajectories across pseudotime between pPROM and sPTL. Pseudotime trajectory UMAPs represent placental cell differentiation in the control, premature preterm rupture of membranes (pPROM), and spontaneous preterm labor (sPTL) groups. The dark blue represents the original starting point of pseudotime, which is scaled as 0, and the yellow represents the tertiary point of pseudotime, which is scaled as 15 in the control group (**A**) and as 20 in the pPROM (**B**) and sPTL (**C**) groups. Boxplots represent variant subtypes of cell clusters (y-axis) that are arranged from bottom to top in ascending order of median pseudotime values (x-axis) in the control (**D**), pPROM (**E**), and sPTL (**F**) groups. The feature plots display all significant differentially expressed genes (adjusted p < 0.05, FDR correction) across pseudotime for the control (**G**), pPROM (**H**), and sPTL (**I**) groups

Trajectory analyses provided potential insights into the differences in disease progression as shown by pseudotime branching, which tracks the transcriptional status and differentiation of cell clusters across conditions [29]. In addition to the differentiation, various mechanisms, including cell turnover, can also be in effect. In pPROM, cell clusters dM2 (*DNAJB1*) and endothelial_fetal (*RRM2*) represented earlier pseudotime cell types, whereas endothelial_lymphatic (*AL357507.1*), mixed immune (*TOP2A*), and T (*THEMIS*) cells appeared at later pseudotime points. Unlike pPROM, sPTL exhibited clear branching points, especially from the earliest pseudotime cell type, CTB (*MKI67*), leading to two distinct branches: one involving the differentiation of CTB to VCT cells, and the other involving STB and endothelial cells, which were of the latest pseudotime (Fig. 3A–F).

Significant changes in DEGs were observed across pseudotime. In the control group, ISG15 ubiquitin-like modifier, TTLL10 antisense RNA 1, stromal cell-derived factor 4, matrix remodeling–associated 8, and aurora kinase A–interacting protein 1 showed differential expression over pseudotime (Fig. 3G). In the pPROM group, genes such as tumor necrosis factor receptor superfamily members 18 and 14, potassium voltage-gated channel subfamily A regulatory beta subunit 2, matrix remodeling-associated 8, Rho guanine-nucleotide exchange factor 16, and ERBB receptor feedback inhibitor 1 were highly expressed in many early pseudotime clusters. These clusters included dM2 (*DNAJB1*), epithelial cells (*RRM2*), endothelial_fetal (*ITGA2*), and various trophoblasts such as EVT3 (*LINC00511*), EVT3 (*GPR78*), VCT (*ATP13A4*), EVT1 (*FLT1*), CTB (*AC007368*), and STB (*MYH14*) (Fig. 3H). Similar to pPROM, sPTL also displayed significant DEG with six key genes identified: protein kinase c zeta, pleckstrin homology and RhoGEF domain containing G5, phosphatidylinositol-4,5-bisphosphate 3-kinase catalytic subunit delta, eukaryotic translation initiation factor 4 gamma 3, podoplanin, and phospholipase A2 group V. *PRKCZ* exhibited consistent expression, with its highest levels in later pseudotime STB, and endothelial (*SPTLC*3) and endothelial_fetal (*ACVRL1*) cells. In contrast, *PLEKHG5*, *PIK3CD*, and *EIF4G3* displayed significant differential gene expression primarily in the dM2 (CR1) cell type (Fig. 3I).

### Cell-to-cell communication signaling

**Fig. 4:**
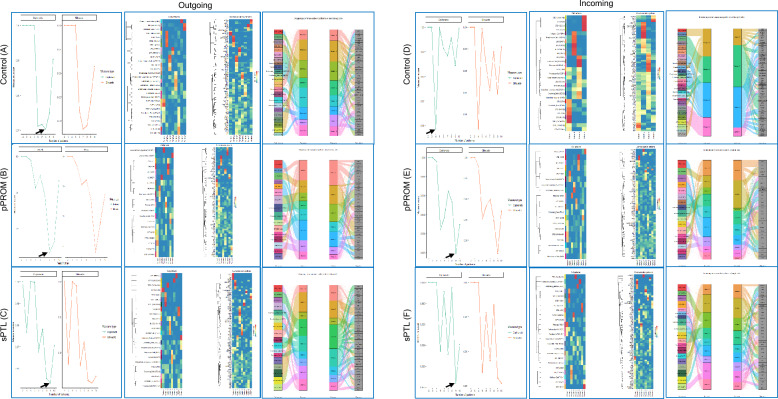
Global cell-to-cell communications. For each group, control (**A**, **D**), premature preterm rupture of membranes (pPROM) (**B**, **E**), and spontaneous preterm labor (sPTL) (**C**, **F**), data involving the outgoing and incoming signaling were collected. The number of outgoing and incoming signals for the control, pPROM, and sPTL groups was determined by selecting the pattern number at the lowest measure score, excluding the final point, from the cophenetic and silhouette indexes, which, respectively, measure how well the clustering matches the original data, the consistency of clustering, and the separation between clusters [30] Arrows point to the value selected. A corresponding heatmap shows the cell and communication pathway patterns and their contributions and a Sankey diagram, a flow diagram in which the arrow width is proportional to the quantity (gene expression) to depict changes over time or hierarchy between nodes and presents the increase or the decrease of data elements in two or more time points [27], shows the communication patterns and signaling pathways of secreting cells and target cells

We employed NMF to identify and categorize cell-to-cell communication signals within the pPROM and sPTL groups, comparing them to a control group. NMF reduces dimensionality and obtains cophenetic and silhouette indexes, which help assess clustering quality and consistency within RNA sequencing data [30]. In total, pPROM samples exhibited eight outgoing signals and nine incoming signals, whereas sPTL samples demonstrated nine outgoing and incoming signals. By contrast, the control group displayed eight outgoing and four incoming signaling patterns (Fig. 4A, D).

Within the control group, many clusters of the same cell type exhibited the same cell signaling pattern. Among the outgoing signals, EVT cell types followed pattern 1, with pathways such as angiopoietin-like (ANGPTL) and pleiotrophin (PTN), whereas STBs exhibited pattern 2, involving activin, periostin, follicle-stimulating hormone (FSH), and thyroid-stimulating hormone (TSH). Pattern 3 was associated with immune cells (dMs, mixed immune cells) that are associated with galectin, CD30, B-cell activating factor (BAFF), and C-X-C motif chemokine ligand 1 (CXCL) (Fig. 4B, C. For incoming signals, STB clusters exhibited pattern 4, involving pathways such as colony-stimulating factor 3 and FSH. Immune cell clusters followed pattern 2, including but not limited to glucocorticoid-induced TNFR-related protein–ligand, BAFF, IL-16, and galectin (Fig. 4E, F).

In contrast to the control group, the pPROM group did not display the same pattern across cell types. In outgoing secreting cells, multiple trophoblast cell types—CTB, STB, VCT, and EVT—were associated with pattern 1, whereas only immune cells were associated with pattern 2. Patterns 5, 6, and 7 also involved immune cells but shared the same patterns with other cell types such as fibroblasts, CTB, and STB. These patterns consisted of many immune-related pathways, including osteopontin (SPP1), ANGPTL, CD30, CD70, and class 3 semaphorin (SEMA3) (Fig. 4B, C). Similar observations were made in incoming target cells. Pattern 1 comprised a mixture of EVT, STB, and fibroblast cell types. Aside from pattern 2, which was specific to immune cells, patterns 6 and 7 were shared with other cells, including CTB and STB and highlighted a mix of immune and cellular pathways including galectin and chemokine ligand (CCL) (Fig. 4E, F).

In the sPTL group, aside from pattern 2 (specific to STB1 cells), most trophoblast cell types exhibited unique patterns. Among the few immune cell types available, mixed immune cells and endothelial cells of maternal origin shared pattern 9, whereas other immune cells, such as dMs and Hofbauer cells, followed pattern 4, involving immune-related pathways such as SPP1, IL-10, and CXCL. We noticed similar trends in the incoming signals, whereby individual trophoblast clusters either exhibited their pattern or were grouped within patterns of the same cell type, such as in patterns 1, 2, 4, and 5. Pattern 3 consisted solely of immune cell clusters, which showed strong contributions from the IL-16 pathway (Fig. 4E, F). Overall, the global cell-to-cell analysis highlighted that incoming and outgoing signals are cell- and condition-specific.

**Fig. 5:**
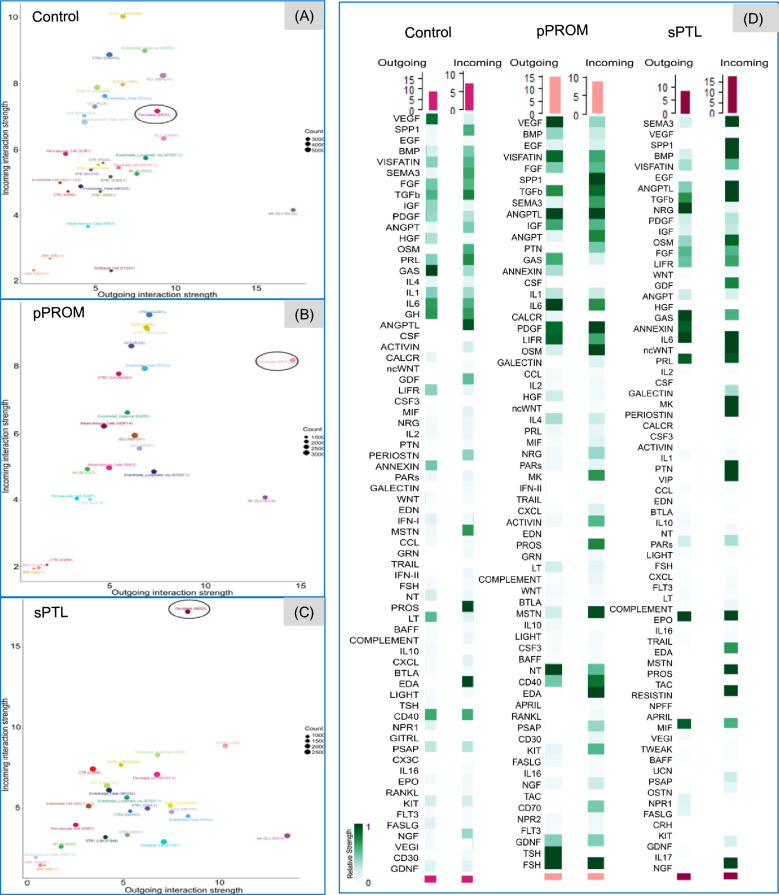
Signaling of cell-to-cell dialogues. Scatterplots of all cell types plotted, with the x-axis representing the signaling strength of outgoing cell-to-cell dialogues and the y-axis showing that of the incoming interaction of cell-to-cell dialogues which cross premature preterm rupture of membranes (pPROM) and spontaneous preterm labor (sPTL), when compared to the control group (**A–C**). The biological function of a specific cell to be incoming or outgoing signaling in a cell-to-cell dialogue may vary on the pathophysiological condition. For example, the circled fibroblast (*MEG3*) had an outgoing signal whose strength was at (x = 15, y = 8) approximately in the pPROM group, whereas it had an incoming signal strength at (x = 8, y = 17) in the sPTL group. Gene expression of various transcripts involved in cell-to-cell dialogues, among all cell clusters in the control, pPROM, and sPTL groups are presented with a heatmap (**D**)

Further analysis focused on fibroblast cell clusters (*MEG3*), in which clear differences in signaling strengths were observed across the control, pPROM, and sPTL groups (Fig. 5A–C). In the control group, outgoing fibroblast signals were dominated by vascular endothelial growth factor (VEGF) and growth arrest–specific (GAS) signaling, suggesting a baseline for maintaining the fetal membrane integrity, angiogenesis, and cell migration [31, 32]. In pPROM, there was an upregulation in VEGF, visfatin, transforming growth factor beta (TGFβ), ANGPTL, interleukin 6 (IL6), platelet-derived growth factor (PDGF), leukemia inhibitory factor receptor, neurotrophin, TSH, FSH, implying active signaling processes associated with tissue repair in response to inflammation and immune response, and hormone regulation [33–39]. In sPTL, neuregulin, GAS, IL6, prolactin (PRL), erythropoietin (EPO), and macrophage migration inhibitory factor signaling were found at high levels with absent VEGF signaling. Like the pPROM condition, the sPTL condition shows upregulated outgoing signaling associated with immune and tissue modulation and hormones [40–45] (Fig. 5D).

For the control condition’s incoming signaling to the fibroblast cluster, patterns of higher displayed ANGPTL, protein S (PROS), and ectodysplasin A (EDA) suggested a baseline of angiogenesis, cell cycle regulation, lipid regulation, and ectoderm development [46–48]. In pPROM, we observed SPP1, ANGPTL, PDGF, oncostatin M, myostatin, CD40, EDA, and FSH signaling, which are involved in inflammation, trophoblast invasion, growth factor, and immune regulation [49–52]. Lastly, sPTL exhibited a diverse range of incoming signaling patterns, including SEMA3, SPP1, bone morphogenetic protein (BMP), ANGPTL, TGFβ, non-canonical WNT (ncWNT), PRL, IL2, midkine, periostin, PTN, EPO, EDA, PROS, resistin, and nerve growth factor, which suggests roles in inhibition of angiogenesis, axon guidance, tissue development, inflammation, trophoblast differentiation, implantation success, and cellular responses [33, 53–59] (Fig. 5D).

### Cell dialogues: immunocyte to trophoblast in pPROM vs. trophoblast to immunocyte in sPTL

**Fig. 6:**
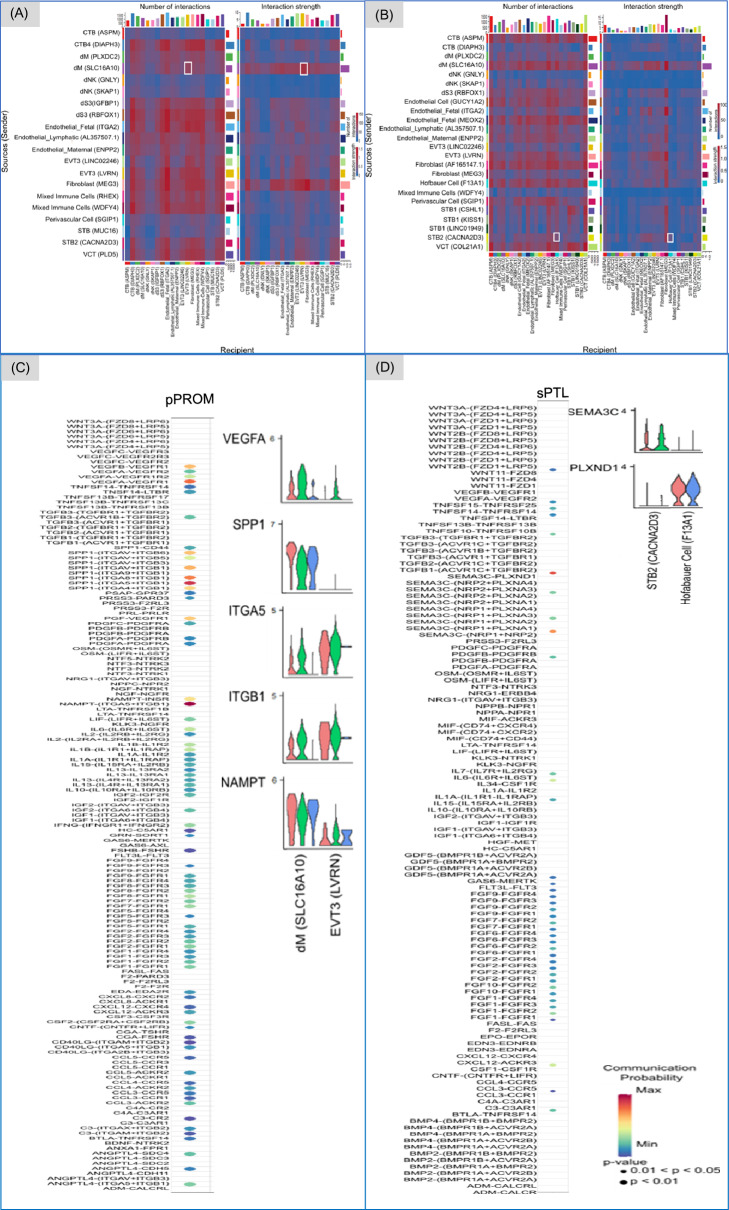
Cell interactions between immunocytes and trophoblasts. White boxes present cell-to-cell interactions of ligand-receptor pairs between immunocyte dM (*SLC16A10*), which functions as the ligand, and trophoblasts EVT3 (*LVRN*), which act as the receptor in the incoming signaling in the clinical condition of premature preterm rupture of membranes (pPROM) (**A**). Similarly, for the outgoing signaling of cell-to-cell interactions, trophoblasts STB2 (*CACNA2D3*) are the ligand, and immunocyte Hofbauer cells (*F13A1*) are the receptor in spontaneous preterm labor (sPTL) (**B**). The dot plots present the communication probability and p-values of specific ligand-receptor pairs involved, and the corresponding violin plots present the transcripts that have been differentially expressed in ligand-receptor (**C, D**)

To explore cell-to-cell communication in sPTB, the interaction between dMs (*SLC16A10*) and the EVTs (*LVRN*) was assessed in the pPROM condition (Fig. 6A), whereas the interaction between STB2 (*CACNA2D3*) and fetal macrophages and Hofbauer cells (*F13A1*) was measured for the sPTL condition (Fig. 6B). We selected interactions involving EVTs in pPROM and STBs in sPTL due to the prominence of these specific cell types within each condition. In pPROM, the interaction from dMs to EVTs was chosen since the dot plot illustrating outgoing and incoming signal interaction strength, indicated strong outgoing signals from dMs (*SLC16A10*) and moderate but highly receptive incoming signals in EVTs (*LVRN*) (Fig. 5B). In the sPTL condition, we prioritized STB (*CACNA2D3*) because it exhibited the strongest outgoing signaling among STB clusters (Fig. 5C). Hofbauer cells (*F13A1*) were selected due to their essential role in supporting STB function within the chorionic villi.

The highest communication probabilities in the pPROM condition were observed in the VEGF, SPP1, nicotinamide phosphoribosyl transferase, TGFβ, CSF, CCL, and the epidermal growth factor signaling pathways (Fig. 6C). These pathways collectively support the intricate balance of tissue homeostasis, immune responses, and angiogenetic repair mechanisms, highlighting their critical roles in maintaining and restoring cellular and tissue integrity [26, 33, 60–64].

In contrast, only two pathways exhibited high communication probabilities in the sPTL condition: semaphorin 3C (SEMA3C) and growth differentiation factor (GDF) (Fig. 6D). GDF signaling, particularly via the GDF15-TGFBR2 interaction, is involved in cell growth and differentiation. SEMA3C signaling, facilitated by interactions such as SEMA3C-PLXND1 and SEMA3C-(NRP1-NRP2), plays a role in inhibiting VEGF-mediated endothelial cell survival, migration, and angiogenesis by altering cytoskeletal dynamics and disrupting endothelial cell adhesion [53, 65, 66]. This inhibition suggests that SEMA3C may regulate blood vessel development within the placental tissue in individuals with sPTL, emphasizing a distinct pathway profile compared to the angiogenic focus observed in pPROM.

## Discussion

The current study closely examined the differences in cell compositions and their transcriptomic profiles between the two clinical features of sPTB, which clearly demonstrate that pPROM is distinct from sPTL despite both being considered premature in clinical management. As evidenced by the UMAPs, EVT clusters were predominantly present in pPROM, whereas STB cell types were more common in sPTL. STBs are known to efficiently transfer nutrients and gases between maternal and fetal tissue while simultaneously restricting the entry of potentially harmful substances and maternal immune cells through intercellular junctions [67]. In contrast, EVTs, particularly the interstitial and endovascular subtypes, are essential for uterine invasion and spiral artery remodeling, crucial processes for establishing adequate maternal–fetal blood flow and nutrient exchange within the placental villi [68]. Hypoxic conditions during early placental development have long been suggested, to elicit cellular responses mediated by hypoxia-inducible factors [69, 70]. These transcription factors significantly impact implantation and placentation processes, as well as increase vascular permeability and angiogenesis within the placental microenvironment [71]. However, although oxidative stress may contribute to the rupture of fetal membranes and exposure to external factors [72], there is currently no research directly connecting hypoxia to pPROM. Nonetheless, it is implied that initial spiral arterial remodeling is likely regulated by immune cells localized in vessels before EVT involvement [73]. Our findings suggest that the inflammatory microenvironment and oxidative stress can promote the formation of immature EVTs within the placenta, akin to the pathological changes in cervical stromal cells [74].

Our single-cell data support this notion, revealing robust recruitment of immune cells, upregulation of tumor necrosis factor receptor superfamily members, and increased cytokine expression in PTB samples, indicative of inflammation within the placental microenvironment associated with pPROM. Moreover, our analysis identified differential expression of genes such as *ERRFI1* and enrichment of the MAPK pathway, implicating cellular growth and stress responses in shaping the intricate landscape of pPROM pathogenesis. Additionally, our findings suggest a potential role for integrins in mediating the increased invasive properties of EVTs in pPROM, as evidenced by elevated integrin expression levels in our cell-to-cell communication analysis from EVT to dM cell types. There was also an upregulation of VEGF expression, which is consistent with hypoxic conditions [71, 72]. Furthermore, the involvement of TGF-β in regulating trophoblast proliferation, differentiation, and decidual EVT invasion underscores the importance of maintaining a balanced cytokine environment for proper placentation. Dysregulation of TGF-β signaling may lead to aberrant placentation and contribute to pregnancy complications, including pPROM [75]. These results implicate inflammation and oxidative stress as significant contributors to the etiology of pPROM and provide comprehensive insights into its multifaceted pathogenesis.

In the context of sPTL, our study highlights distinct pathophysiological mechanisms compared to pPROM, particularly emphasizing the altered trophoblast composition and cell-to-cell communication profiles. Our analysis reveals a predominant presence of STB in sPTL samples, with EVTs being significantly underrepresented. Despite this, persistent inflammation and a unique immune profile were evident in sPTL, suggesting that immune modulation and inflammatory processes play a role in its pathogenesis. Our single-cell analysis identified an enriched expression of pathways involved in stress responses and tissue remodeling in sPTL samples, including those associated with smooth muscle contraction and vascular regulation. Additionally, the cell-to-cell communication analysis uncovered distinct incoming signaling patterns to fibroblasts, such as SEMA3, SPP1, BMP, ANGPTL, TGFβ, and ncWNT, implicating complex interactions influencing angiogenesis, trophoblast cell life cycle, migration, and tissue remodeling. Furthermore, our analysis revealed the expression of hormones such as prolactin and erythropoietin originating from the outgoing signals of fibroblasts, suggesting the contribution of a complex interplay of signaling pathways to the pathogenesis of preterm labor. The distinct cellular and molecular profiles in sPTL and pPROM reflect the complexity of PTB subtypes and underscore the importance of tailored therapeutic approaches for each condition.

## Conclusion

This study provides a comprehensive single-nucleus transcriptomic analysis of the maternal–fetal interface, revealing key differences in the trophoblast cell composition, gene expression, and molecular interactions between the two major subtypes of sPTB: pPROM and sPTL. Given that preterm birth is a significant cause of mortality and morbidity, our study further emphasizes the necessity of distinguishing between these subtypes to understand their unique etiology and pathophysiology for potential therapeutic targets. We observed notable alterations in trophoblast cell populations, EVT and STB, which may act as critical factors associated with the outcomes of pPROM and sPTL. In addition, we demonstrated that infection- and inflammation-associated pathways play significant roles in these conditions, with pPROM associated with cytokine activation, matrix metalloproteinase induction, and apoptosis, while sPTL involves molecular mechanisms driving uterine contractility and cervical ripening.

Our study also highlights the need for improved molecular diagnostics and prognostic markers for further exploration into the sPTB subtypes. Future studies should expand upon our findings, integrating multi-omics, to provide a comprehensive study of the maternal–fetal interface. In addition to functionally validating key genes and pathways, studies can dive deeper and identify any connections and impacts that pPROM and sPTL may have on fetal neurodevelopment, thereby for novel therapeutic strategies mitigating the risk of preterm birth and its associated complications.

## Supplementary Information

Supplementary Material 1.

Supplementary Material 2.

Supplementary Material 3.

Supplementary Material 4.

Supplementary Material 5.

## Data Availability

All data are available in the main text or the supplementary materials: Fig. S1A–S6B, data files: Table S1–S4.
