# Correction: Single-nucleus RNA sequencing reveals distinct pathophysiological trophoblast signatures in spontaneous preterm birth subtypes

**DOI:** 10.1186/s13578-025-01421-x

**Published:** 2025-06-26

**Authors:** Cherilyn Uhm, Jianlei Gu, Weina Ju, Stephanie Pizzella, Hande Oktay, Joyce Yao-Chun Peng, Sararose Guariglia, Yong Liu, Hongyu Zhao, Yong Wang, Ramkumar Menon, Nanbert Zhong

**Affiliations:** 1 New York State Institute for Basic Research in Developmental Disabilities, Staten Island, NY 10314 USA; 2 School of Public Health, Yale University, New Haven, CT 06520-0834 USA; 3 Department of Obstetrics and Gynecology, School of Medicine, Washington University, St. Louis, MO 63110-1010 USA; 4 Singulomics Corporation, Bronx, NY 10461 USA; 5 The University of Texas Medical Branch at Galveston, Galveston, TX 77555-0144 USA

**Correction to: Cell & Bioscience (2025) 15:1**

10.1186/s13578-024-01343-0.

In Table 1 of this article [1], under the Group sPTL, Sample 1 and Sample 2, the gestational ages 40.4 and 40.2 were incorrect and should have been 36.0.

**Incorrect Table **1:

**Table 1:** Sample number, maternal age, history of pregnancy (Hx pregnancy), and gestational age of control, pPROM, and sPTL pregnancy groups.

Group	Sample Number	Maternal Age	Hx Pregnancy	Gestational Age
Control	1	39	G6P4	39.0
	2	23	G3P2	39.0
	3	29	G4P2	39.0
pPROM	1	30	G4P2	34.3
	2	24	G3P2	33.3
sPTL	1	23	G1P0	40.4
	2	34	G3P1	40.2

**Correct Table** 1:

**Table 1:** Sample number, maternal age, history of pregnancy (Hx pregnancy), and gestational age of control, pPROM, and sPTL pregnancy groups

Group	Sample Number	Maternal Age	Hx Pregnancy	Gestational Age
Control	1	39	G6P4	39.0
	2	23	G3P2	39.0
	3	29	G4P2	39.0
pPROM	1	30	G4P2	34.3
	2	24	G3P2	33.3
sPTL	1	23	G1P0	36.0
	2	34	G3P1	36.0
