# Supplementary material for: Single-nucleus RNA sequencing reveals distinct pathophysiological trophoblast signatures in spontaneous preterm birth subtypes
Source: Cell Biosci. 2025 Jan 7;15:1. doi: 10.1186/s13578-024-01343-0 (PMC11705668; doi:10.1186/s13578-024-01343-0)
Supplement: Supplementary file 1 — Supplementary Material 1. [file 13578_2024_1343_MOESM1_ESM.docx]

Figure S1-A


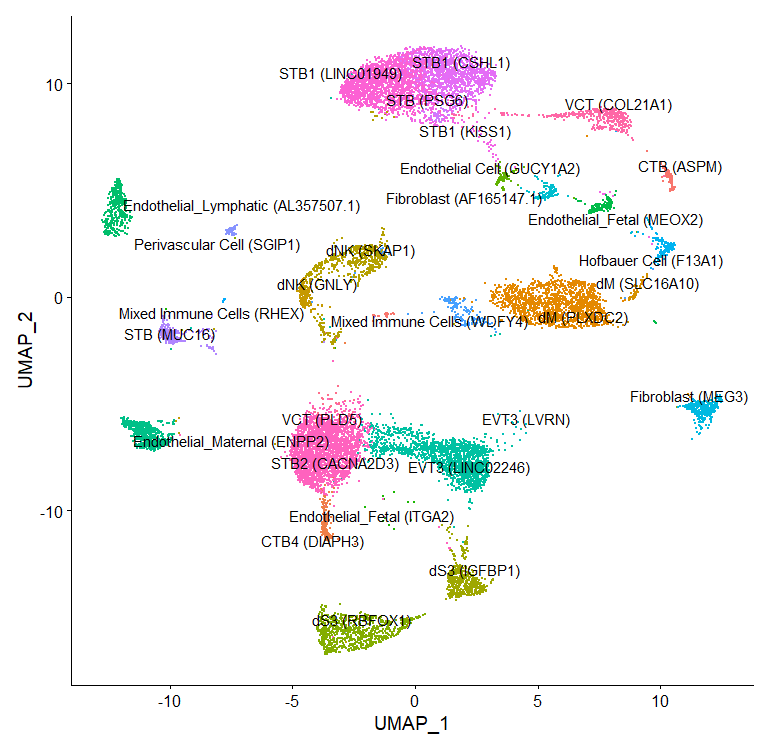


Control


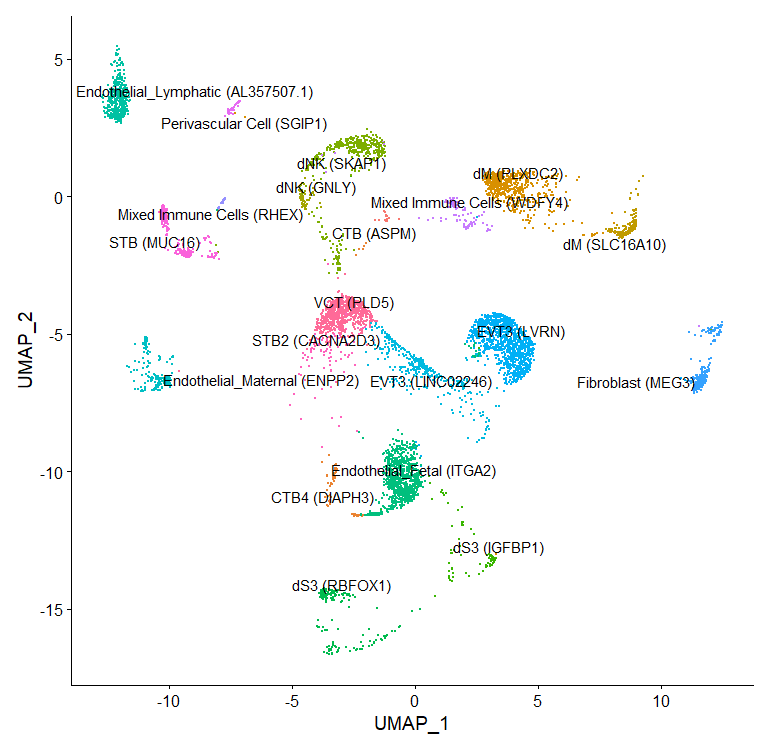

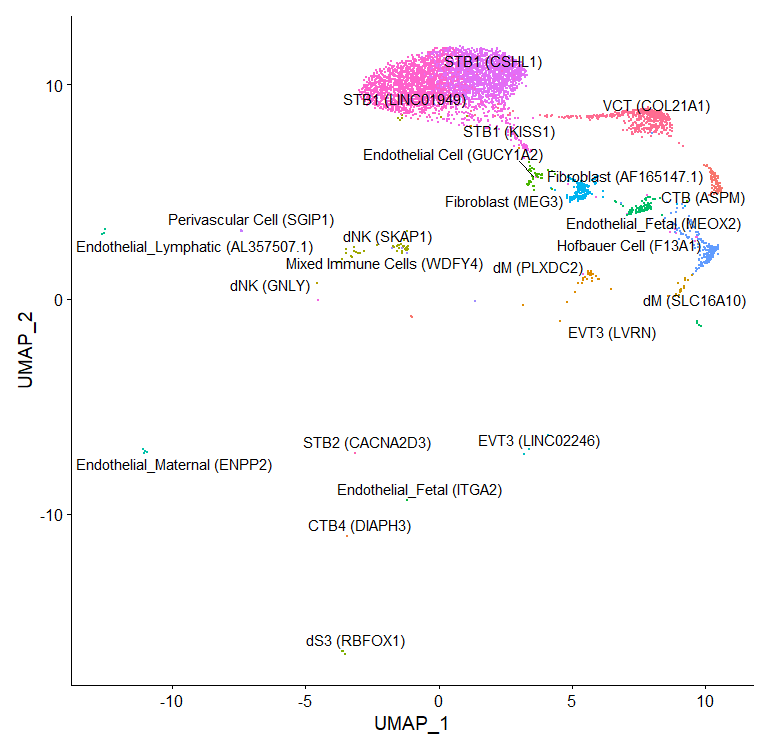

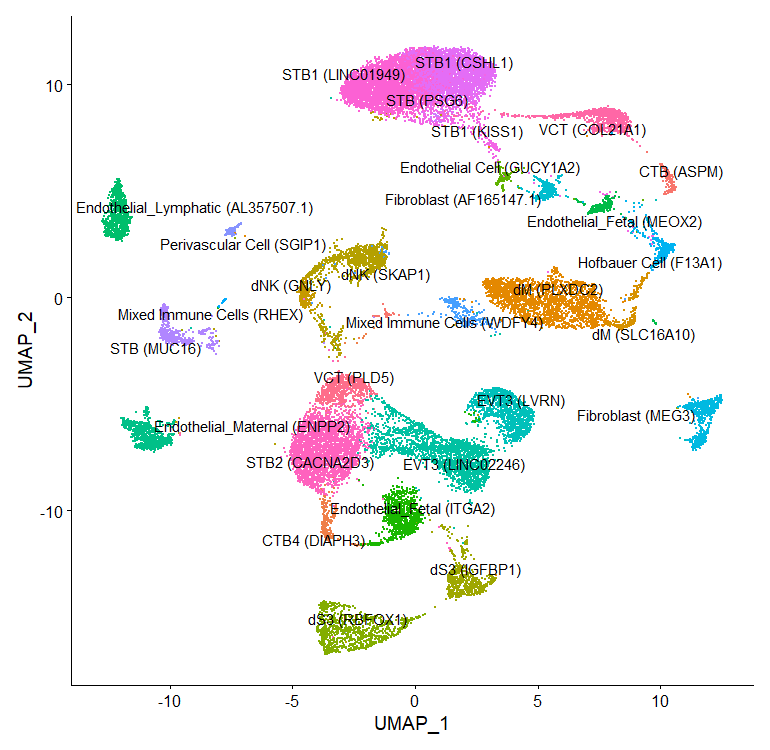


pPROM

sPTL

Integrated

Figure S1-B


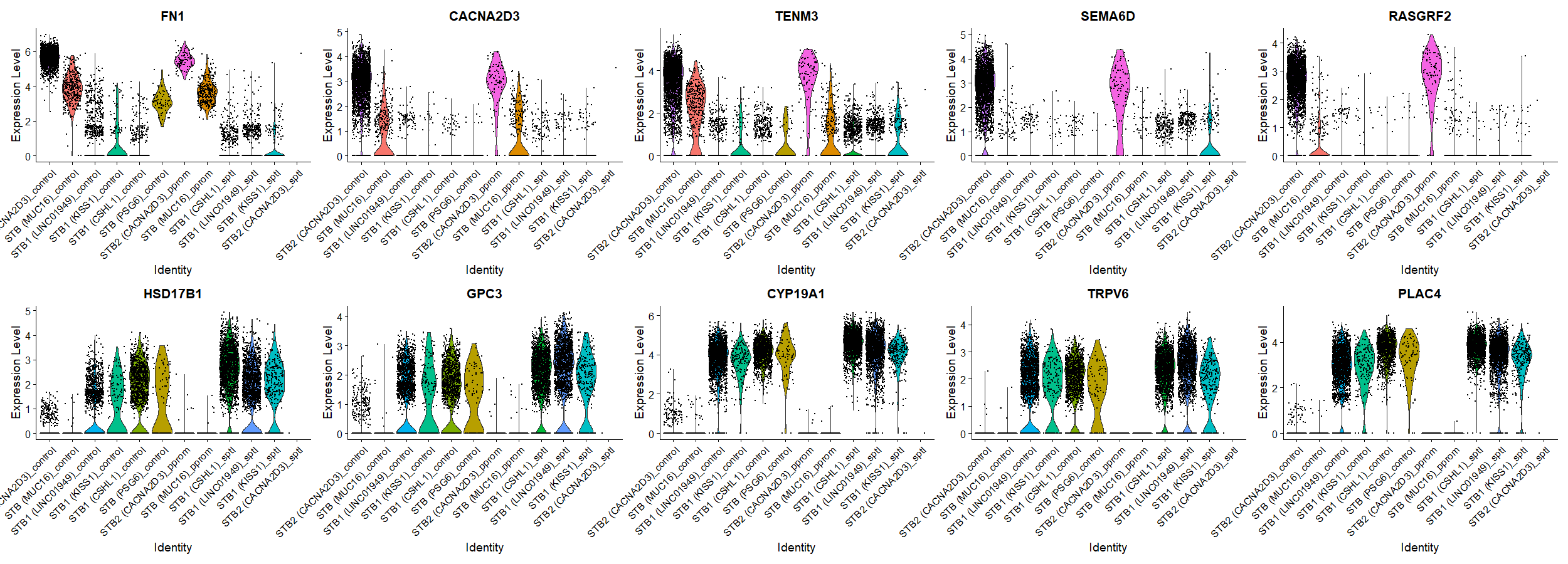

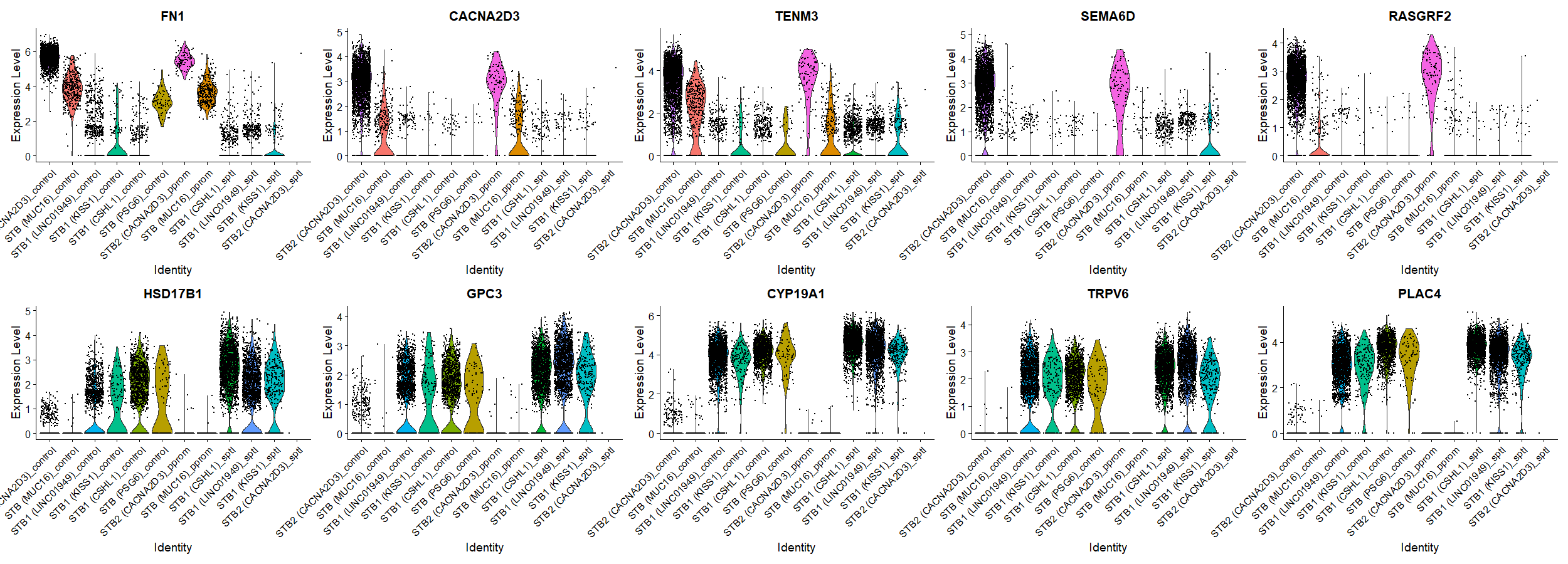


FN1                                 CACNA2D3                               TENM3                              SEMA6D                             RASGRF2

HSD17B1                              GPC3                                  CYP19A1                              TRPV6                                  PLAC4


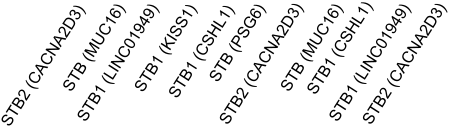


Control    pPROM sPTL


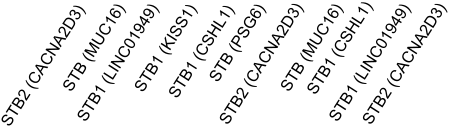

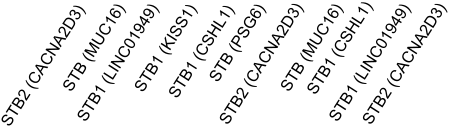

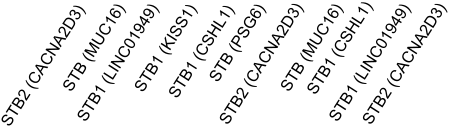

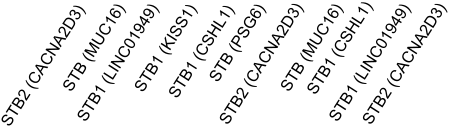

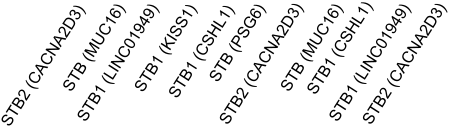

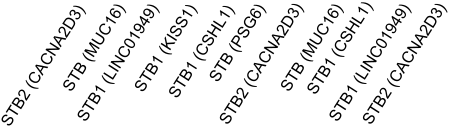

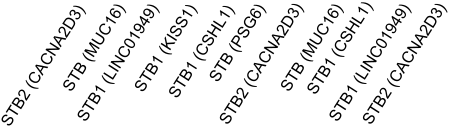

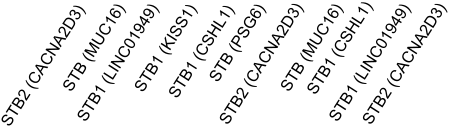

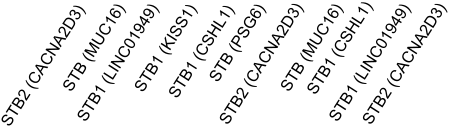


Control    pPROM sPTL

Control    pPROM sPTL

Control    pPROM sPTL

Control    pPROM sPTL

Control    pPROM sPTL

Control    pPROM sPTL

Control    pPROM sPTL

Control    pPROM sPTL

Control    pPROM sPTL


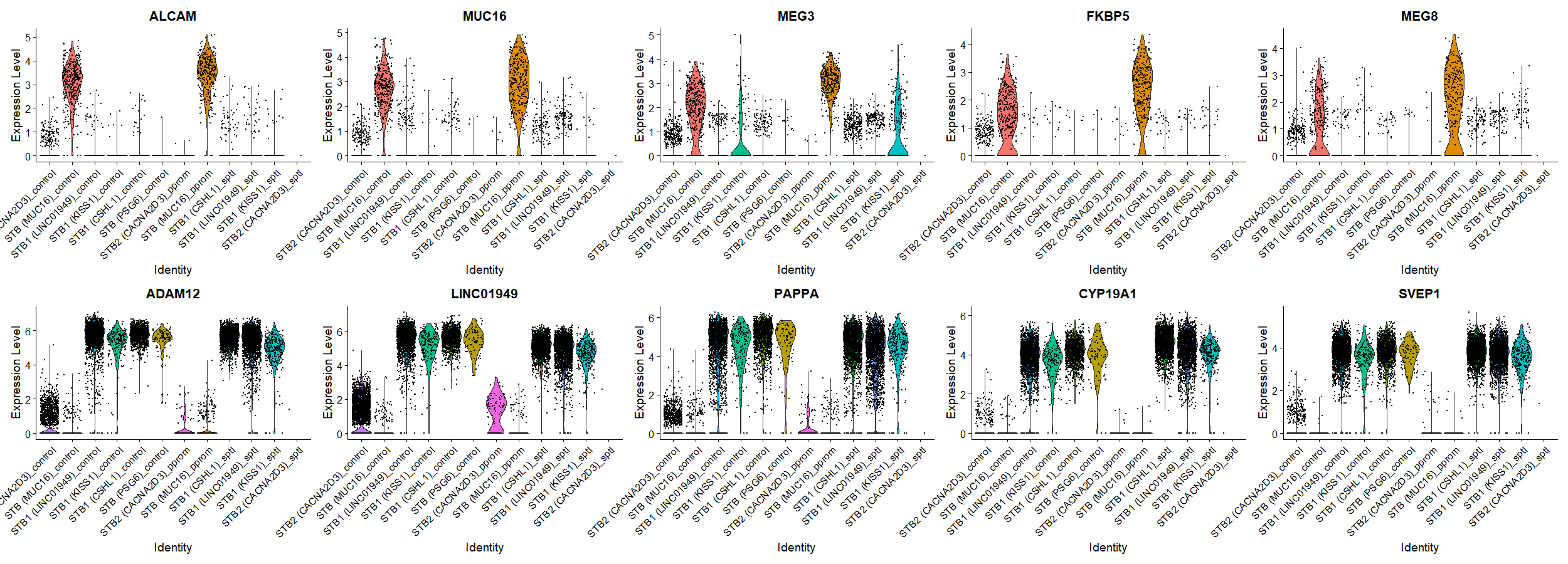

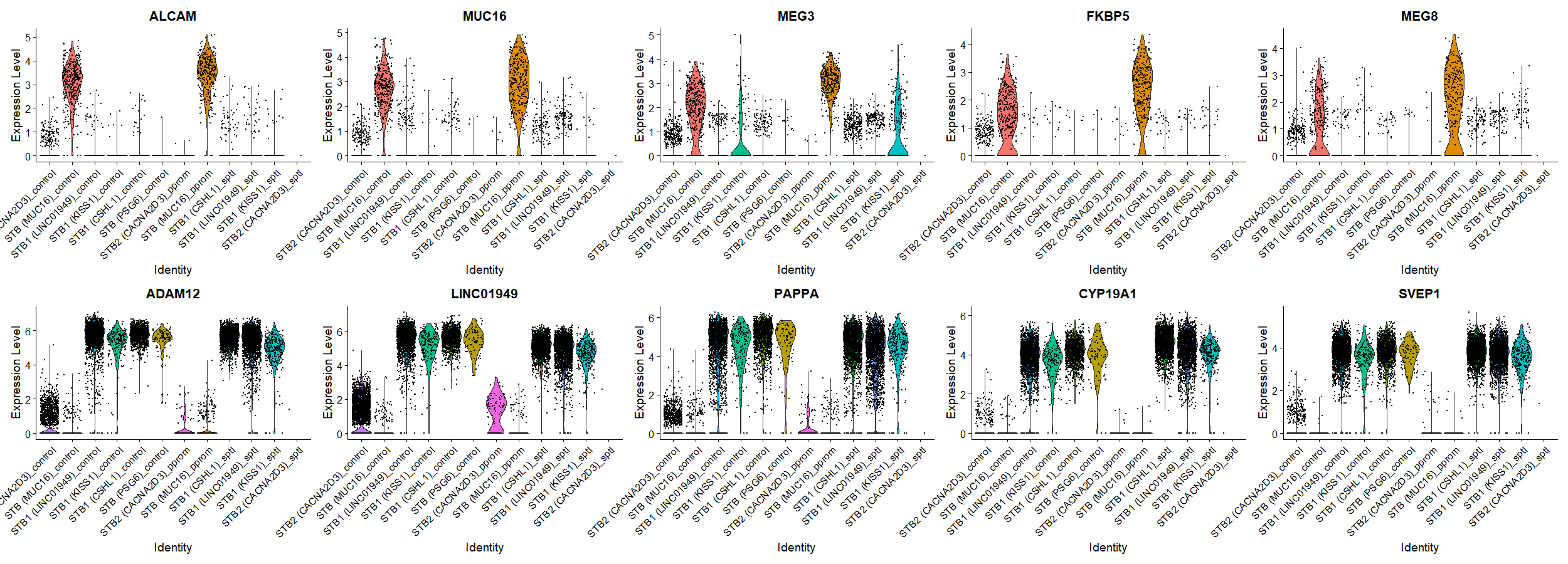

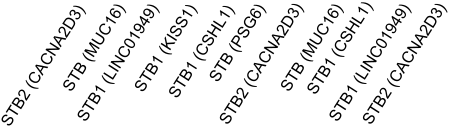

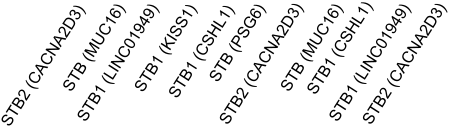

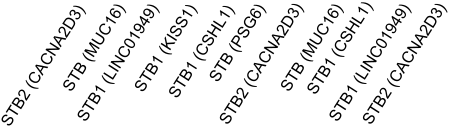

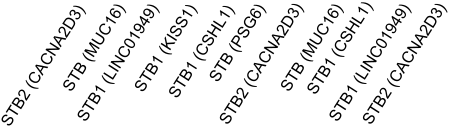

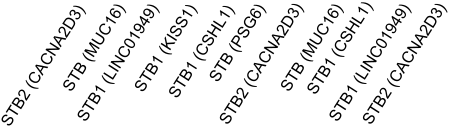


ALCAM                                   MUC16                                   MEG3                                FKBP5                                  MEG8


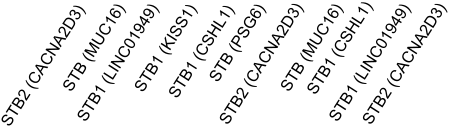

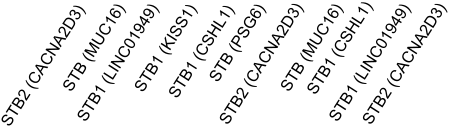

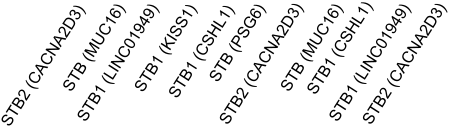

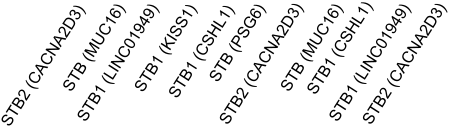

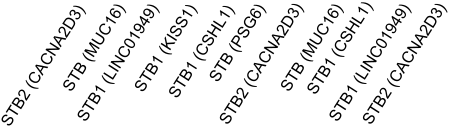


ADAM12                            LINC01949                               PAPPA                              CYP19A1                                SVEP1

Control    pPROM sPTL

Control    pPROM sPTL

 Control    pPROM sPTL

 Control    pPROM sPTL

Control    pPROM sPTL

Control    pPROM sPTL

Control    pPROM sPTL

 Control    pPROM sPTL

 Control    pPROM sPTL

Control    pPROM sPTL


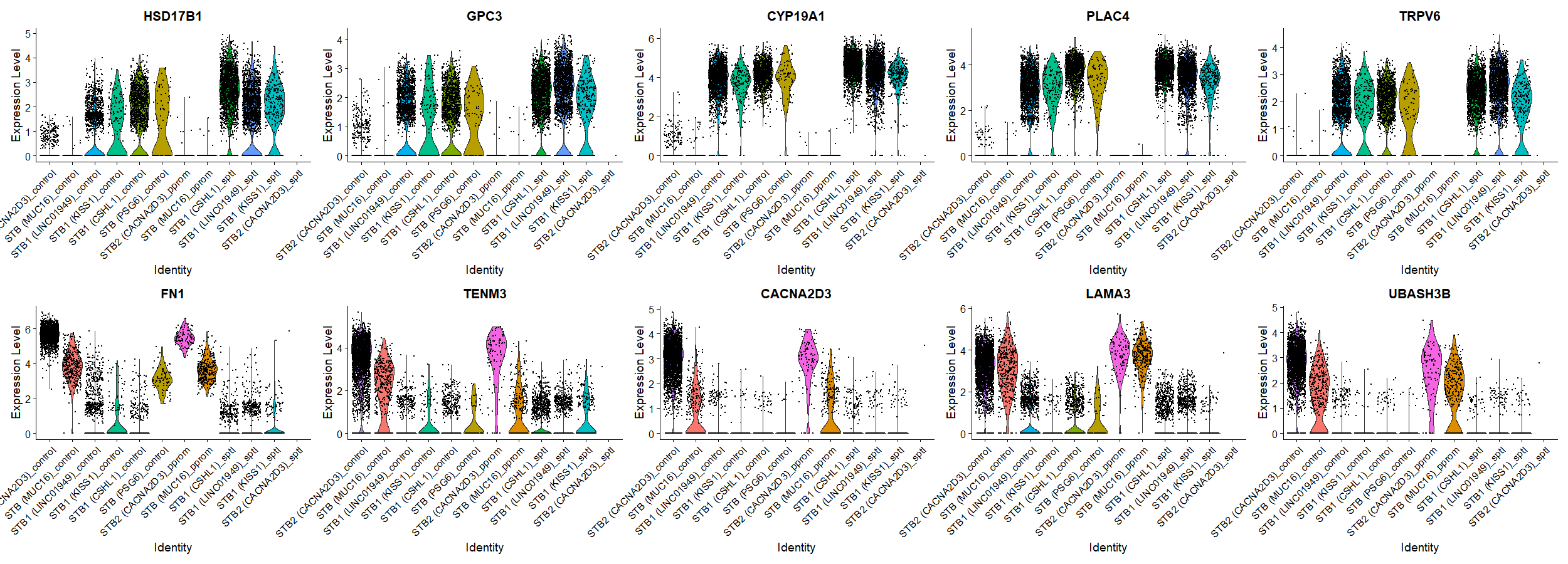

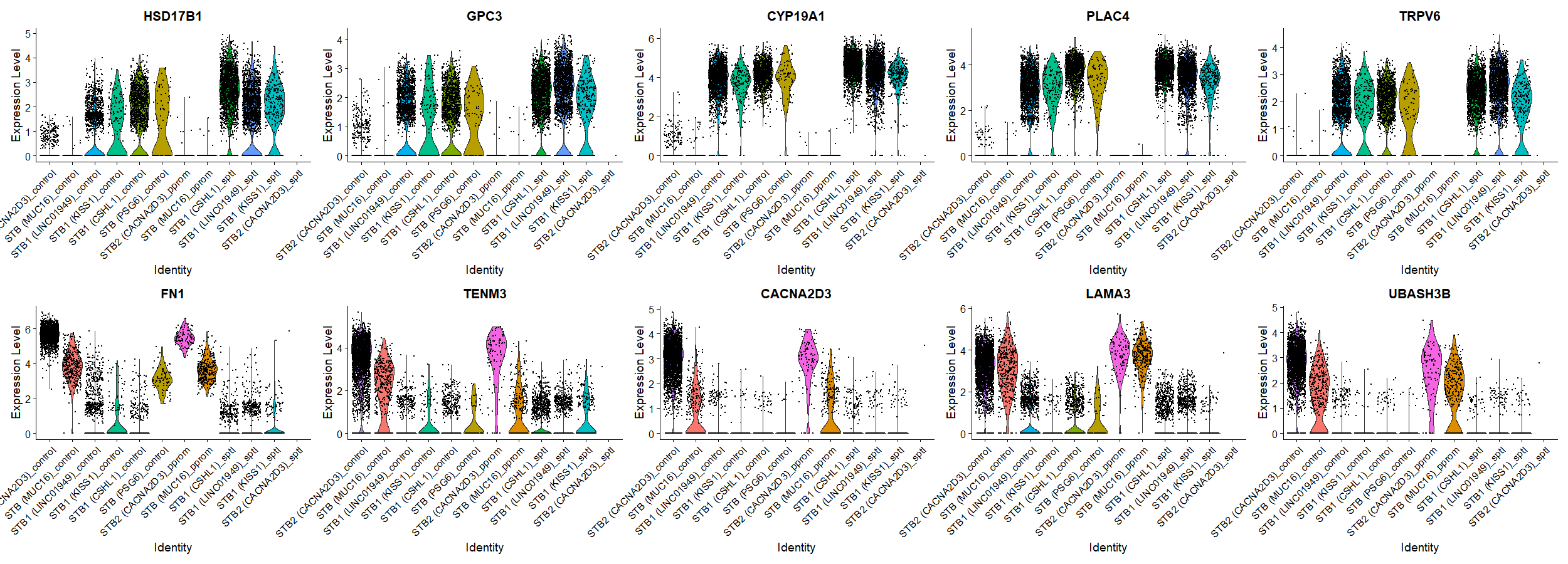


HSD17B1                               GPC3                                  CYP19A1                              PLAC4                         TRPV6

FN1                                    TENM3                             CACNA2D3                            LAMA3                                UBASH3B


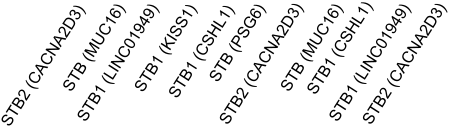

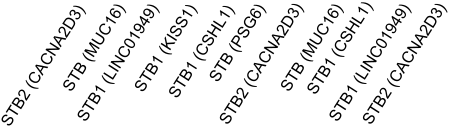

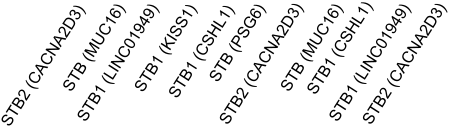

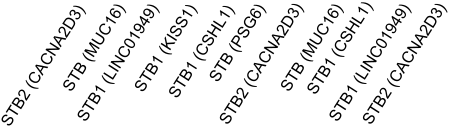

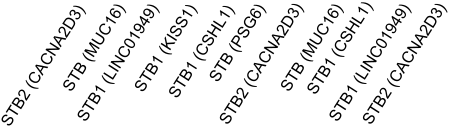

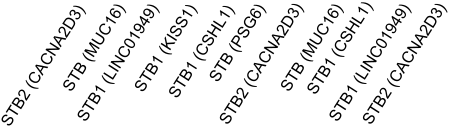

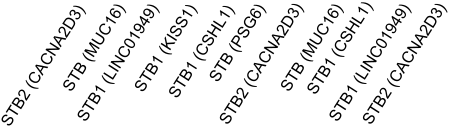

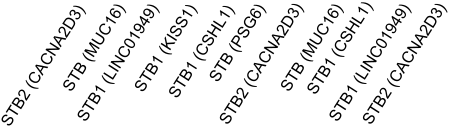

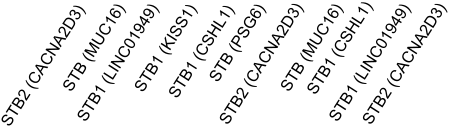

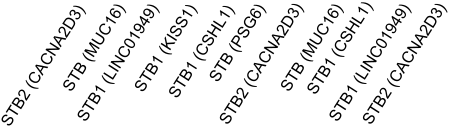


Control    pPROM sPTL

Control    pPROM sPTL

 Control    pPROM sPTL

 Control    pPROM sPTL

Control    pPROM sPTL

Control    pPROM sPTL

Control    pPROM sPTL

 Control    pPROM sPTL

 Control    pPROM sPTL

Control    pPROM sPTL

Control

pPROM

sPTL

STB Cells

Figure S1-C

Control

pPROM

sPTL

EVT Cells


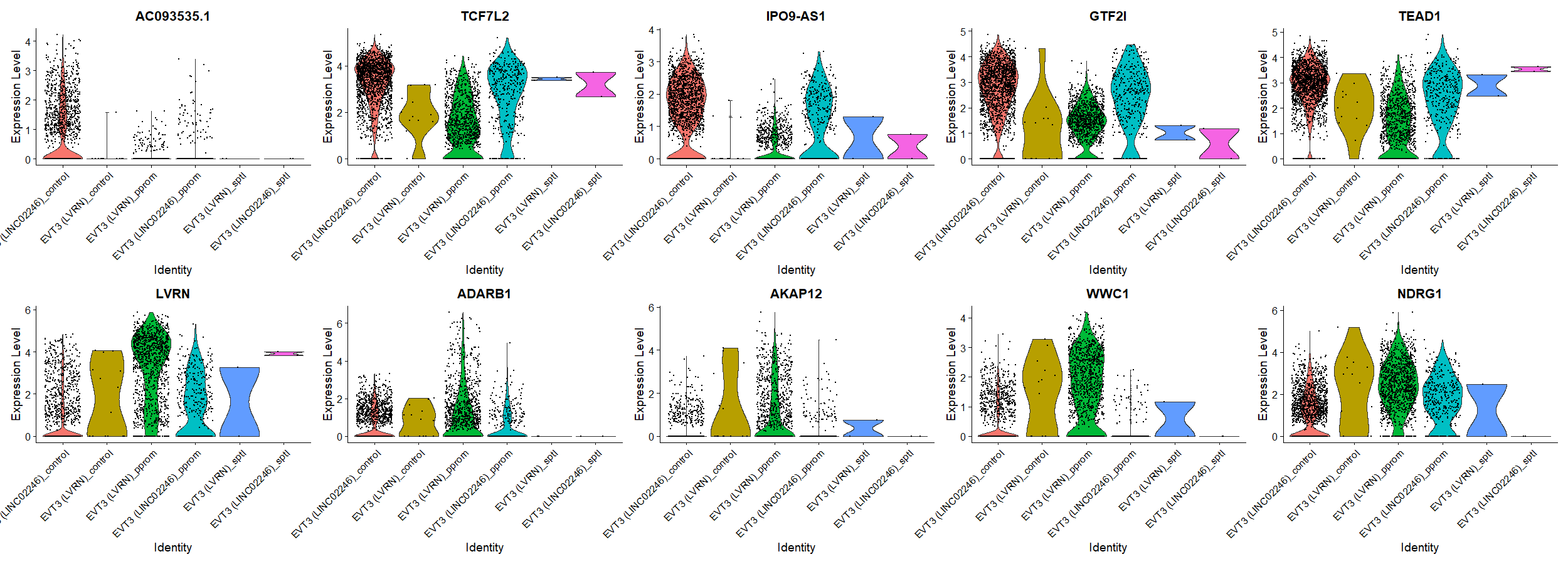

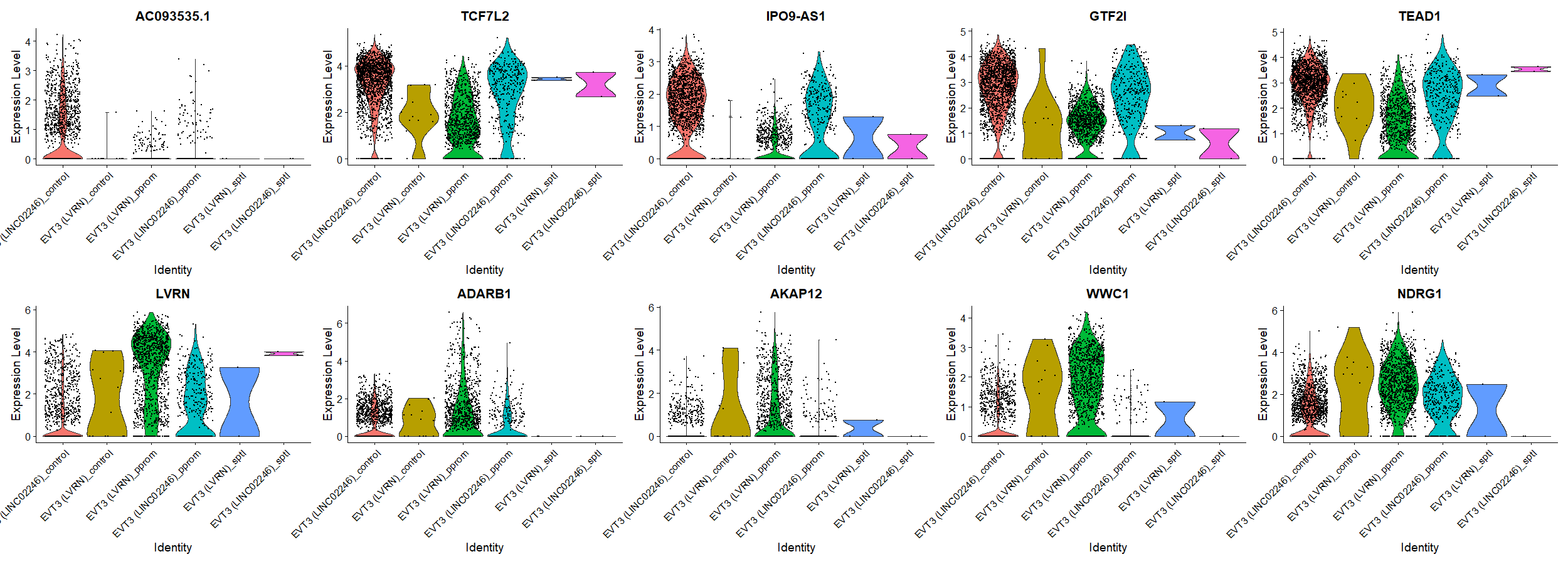

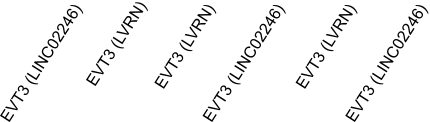


AC093535.1                          TCF7L2                               IPO9-AS1                             GTF2I                                 TEAD1

LVRN                                ADARB1                                AKAP12                            WWC1                                 NDRG1


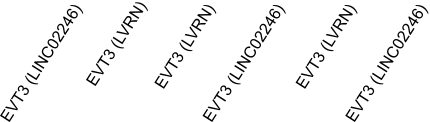


Control   pPROM     sPTL


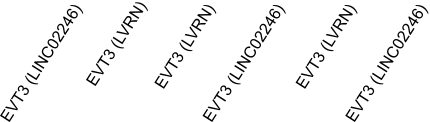

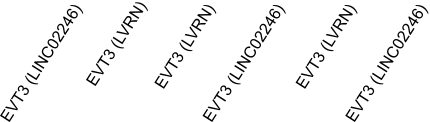

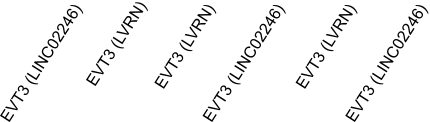

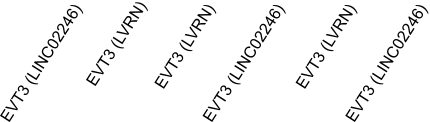

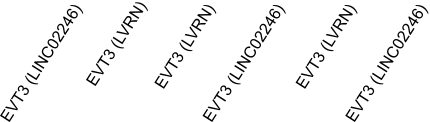

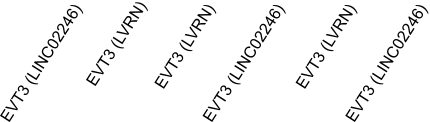

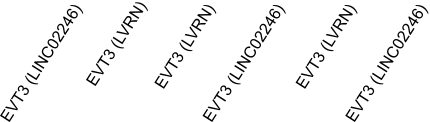

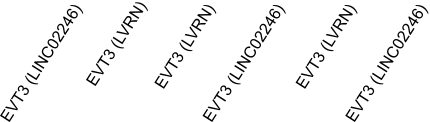


Control    pPROM     sPTL

Control    pPROM     sPTL

Control    pPROM     sPTL

Control    pPROM     sPTL

Control    pPROM     sPTL

Control    pPROM     sPTL

Control    pPROM     sPTL

Control    pPROM     sPTL

Control    pPROM     sPTL

Control    pPROM     sPTL

Control    pPROM     sPTL

Control    pPROM     sPTL

Control    pPROM     sPTL

Control    pPROM     sPTL


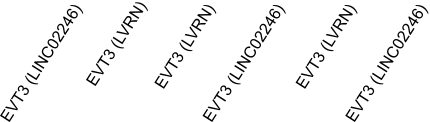

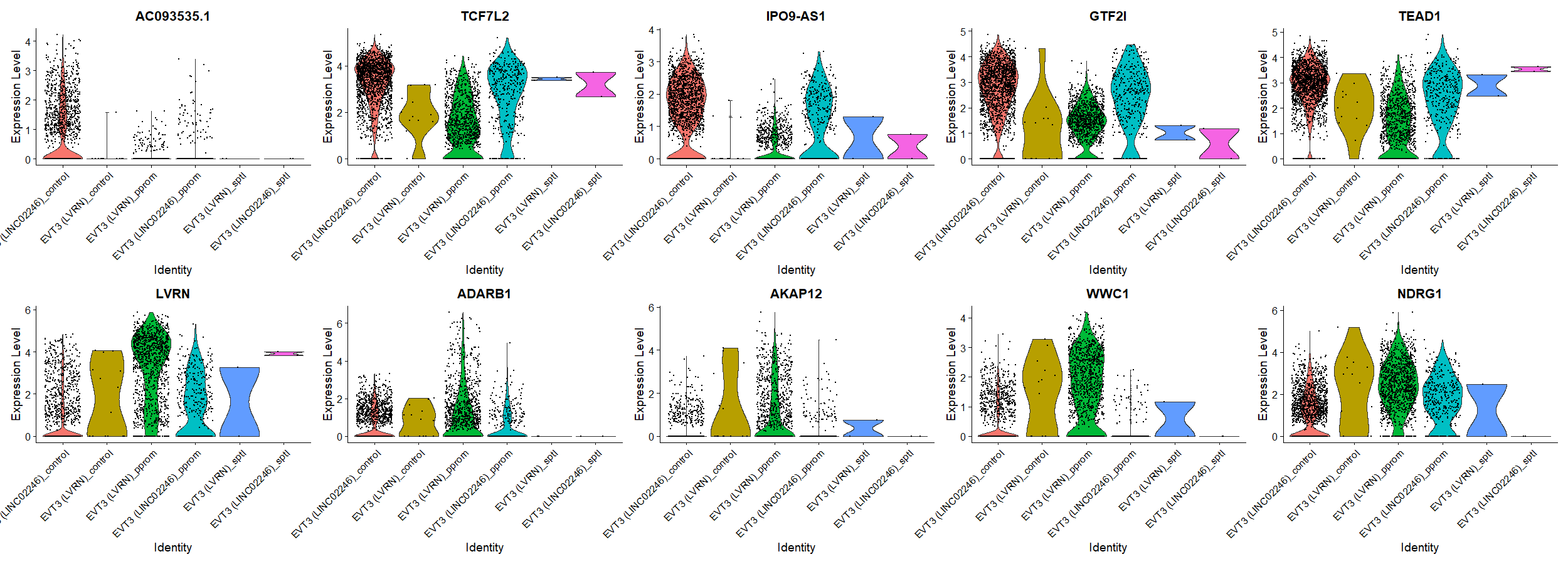

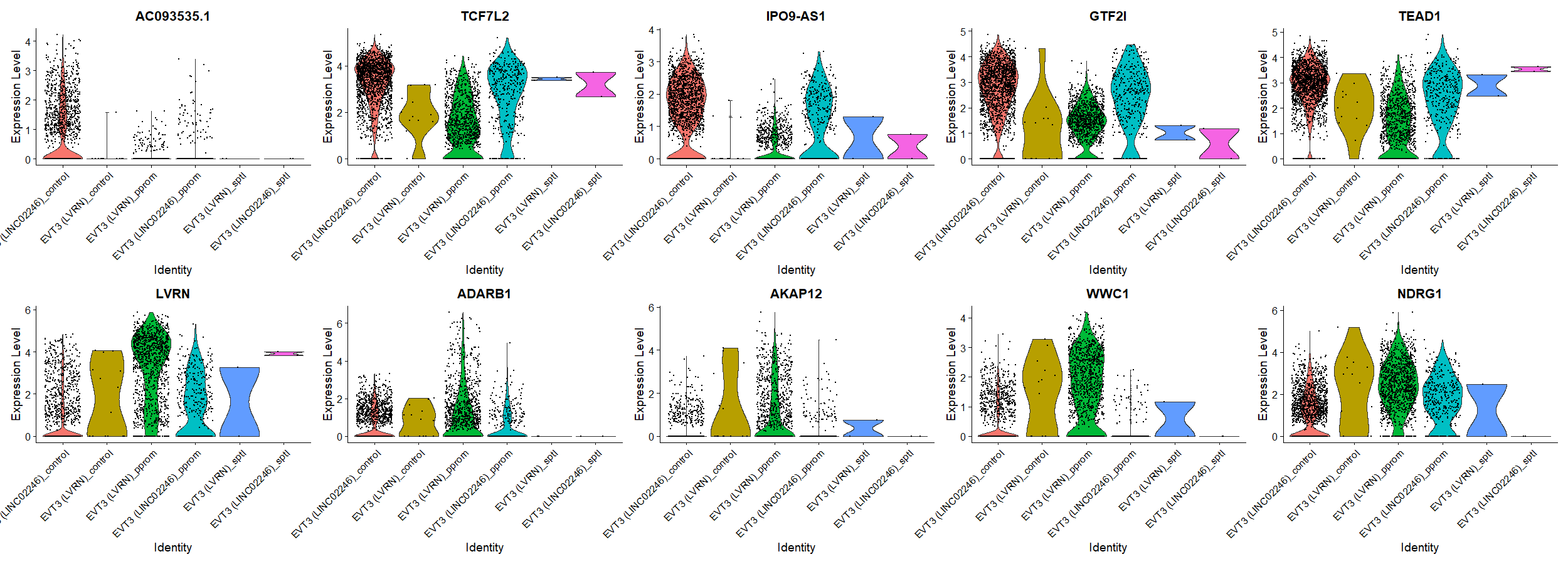

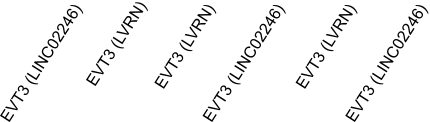

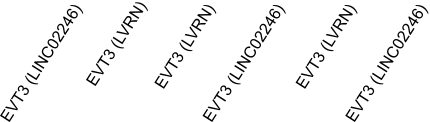

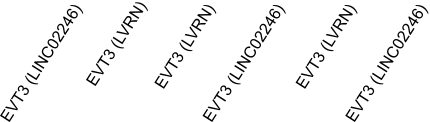

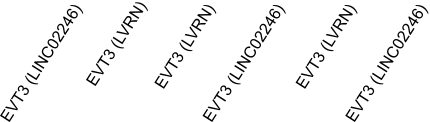

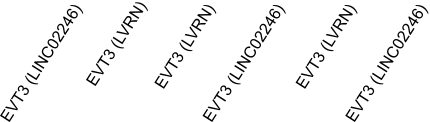

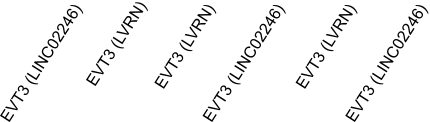

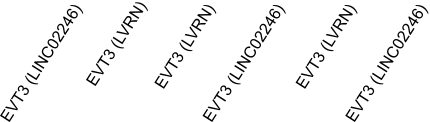

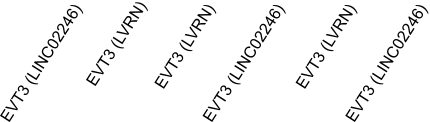

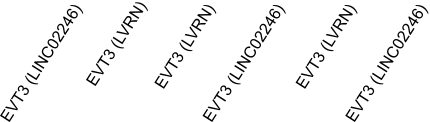


LVRN                                ADARB1                                AKAP12                            WWC1                                 NDRG1

Control    pPROM     sPTL

Control    pPROM     sPTL

Control    pPROM     sPTL

Control    pPROM     sPTL

Control    pPROM     sPTL

AC093535.1                          TCF7L2                               IPO9-AS1                             GTF2I                                 TEAD1

Control    pPROM     sPTL

Control    pPROM     sPTL

Control    pPROM     sPTL

Control    pPROM     sPTL

Control    pPROM     sPTL


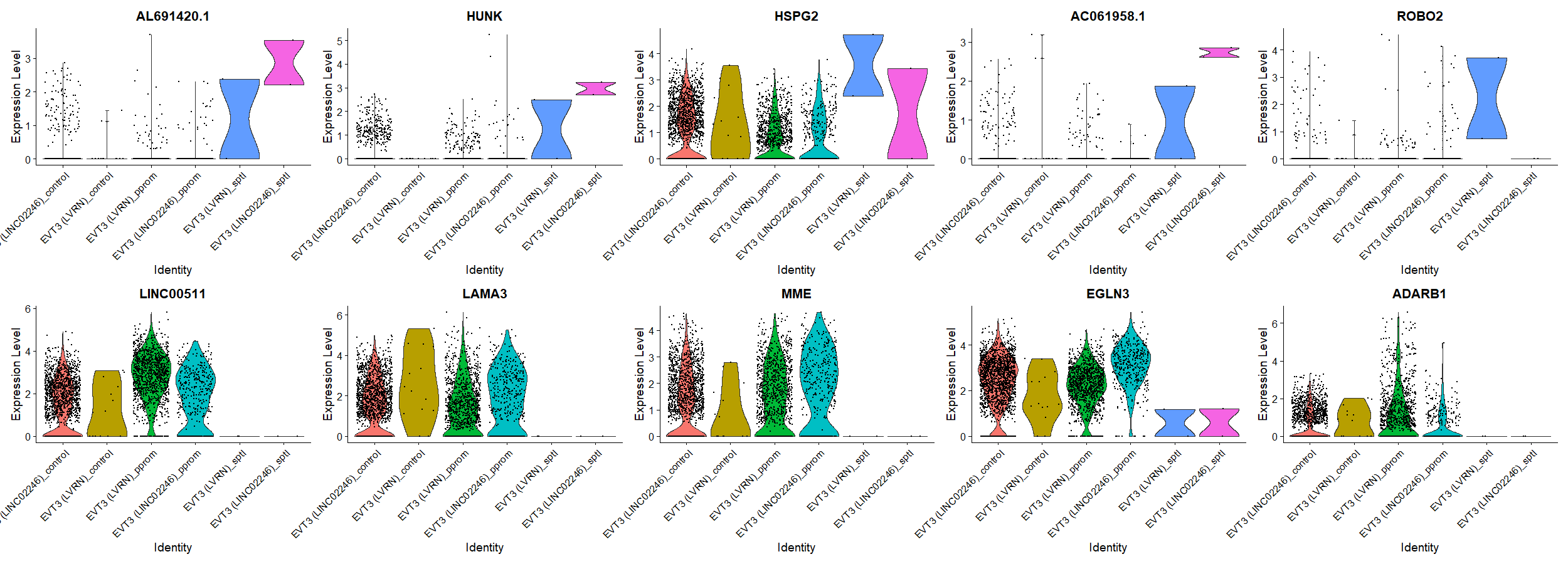

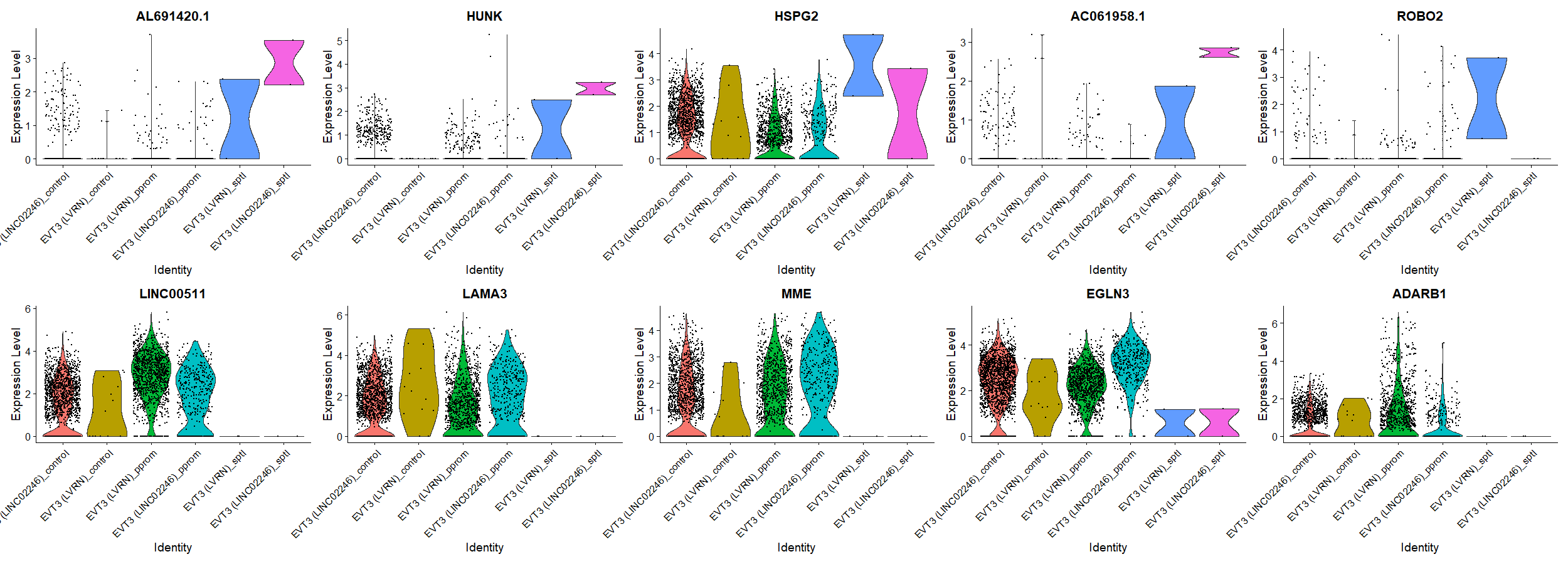


AL691420.1                           HUNK                                  HSPG2                           AC061958.1                            ROBO2

LINC00511                            LAMA3                                  MME                                   EGLN3                              ADARB1


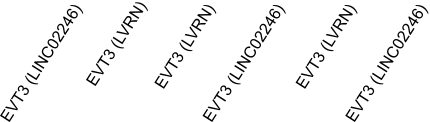

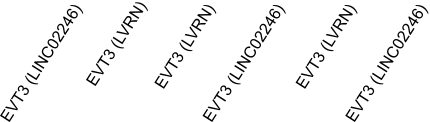

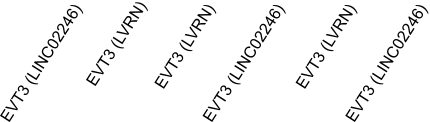

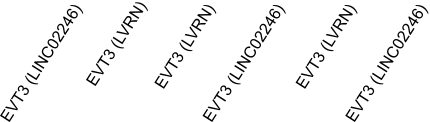

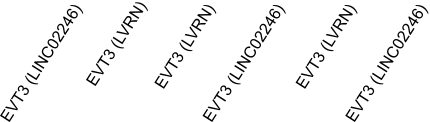

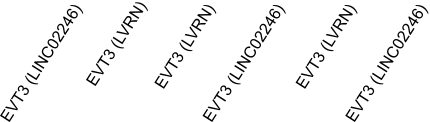

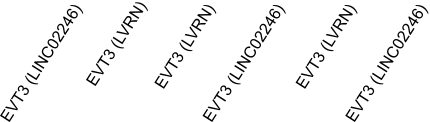

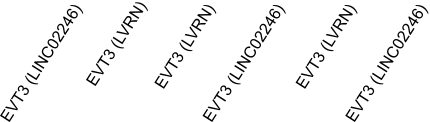

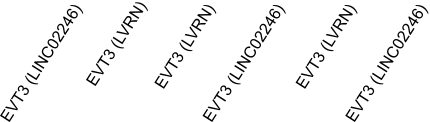

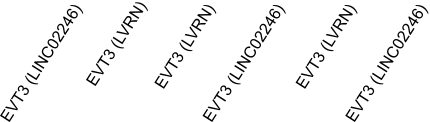


Control    pPROM     sPTL

Control    pPROM     sPTL

Control    pPROM     sPTL

Control    pPROM     sPTL

Control    pPROM     sPTL

Figure S2-A


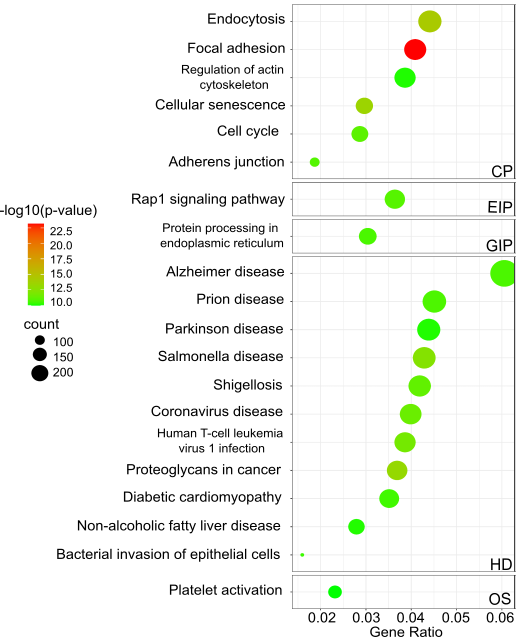


Control


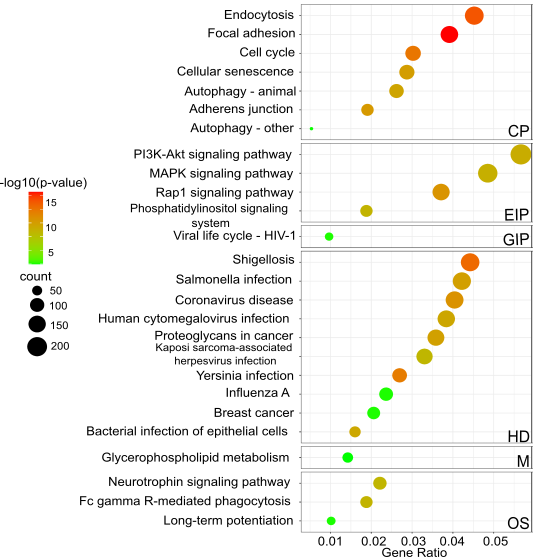


pPROM

sPTL


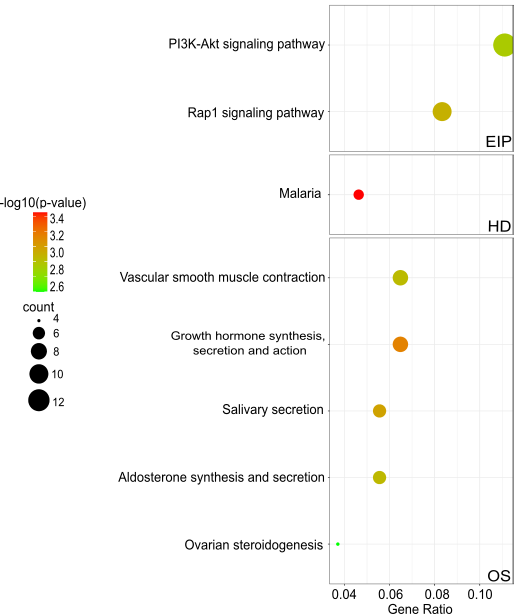


Figure S2-B


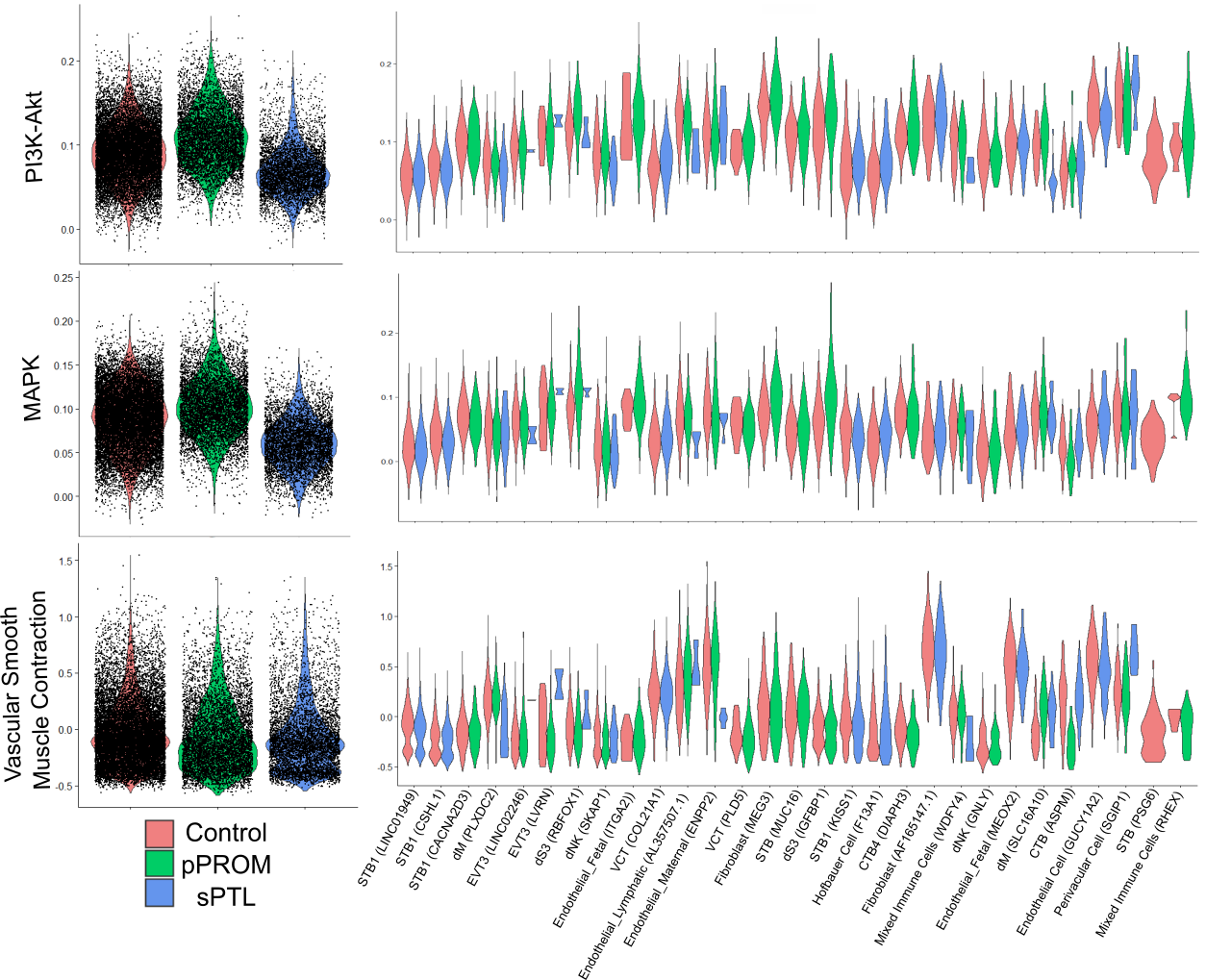

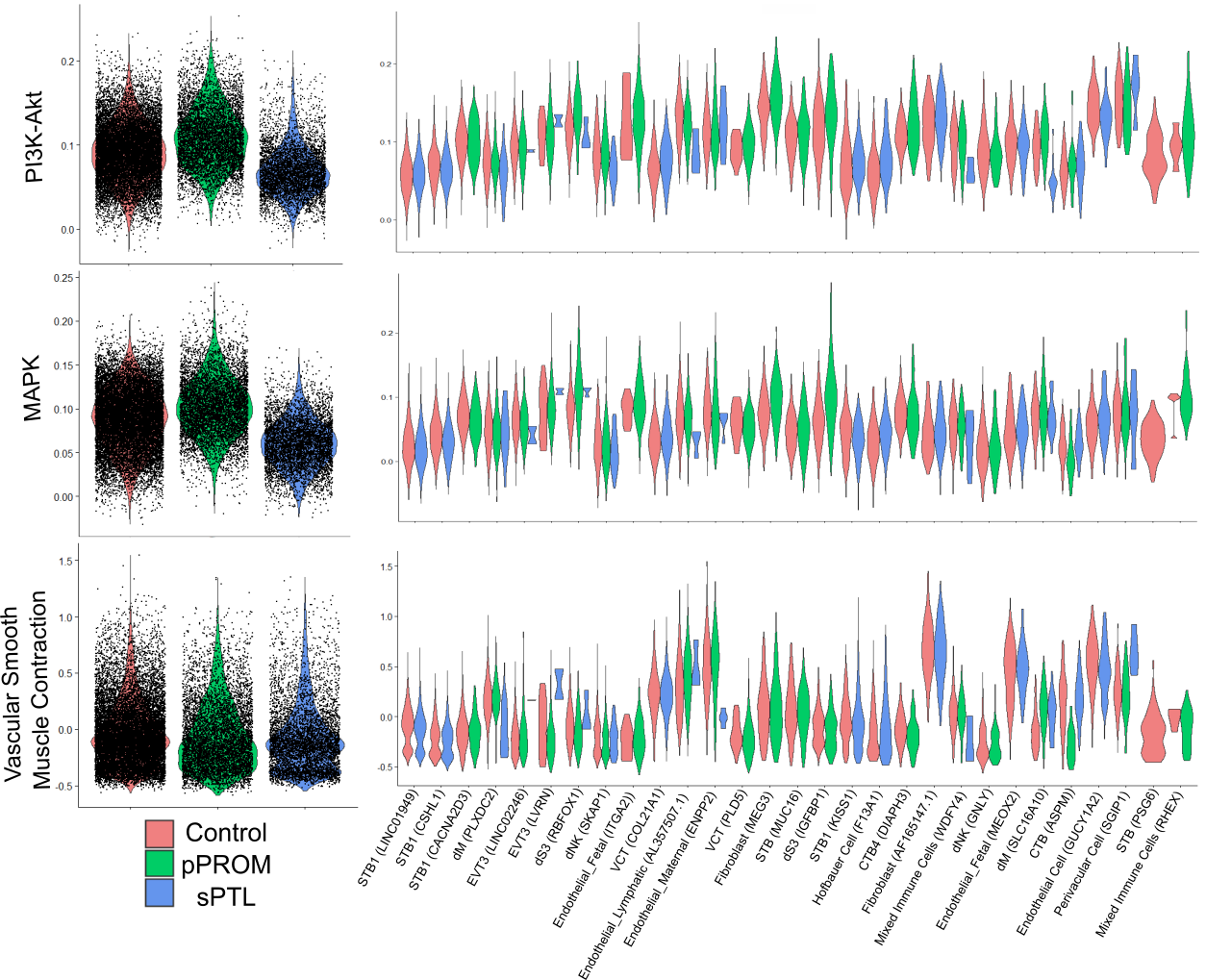


Figure S3-A


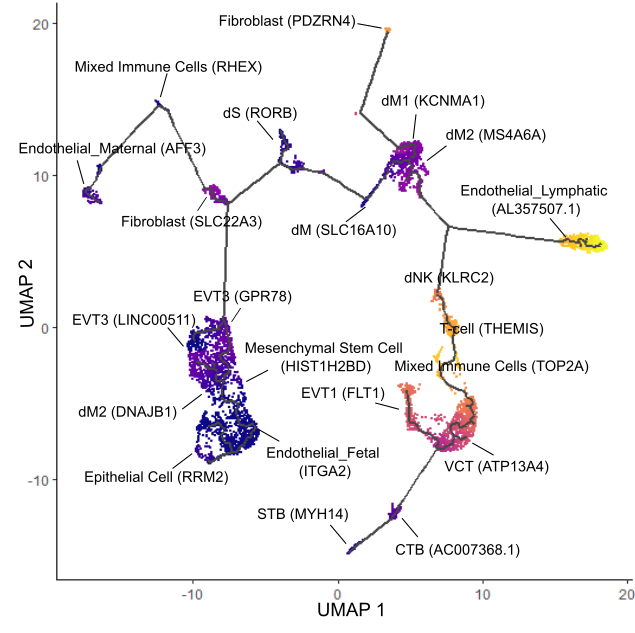

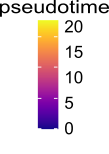

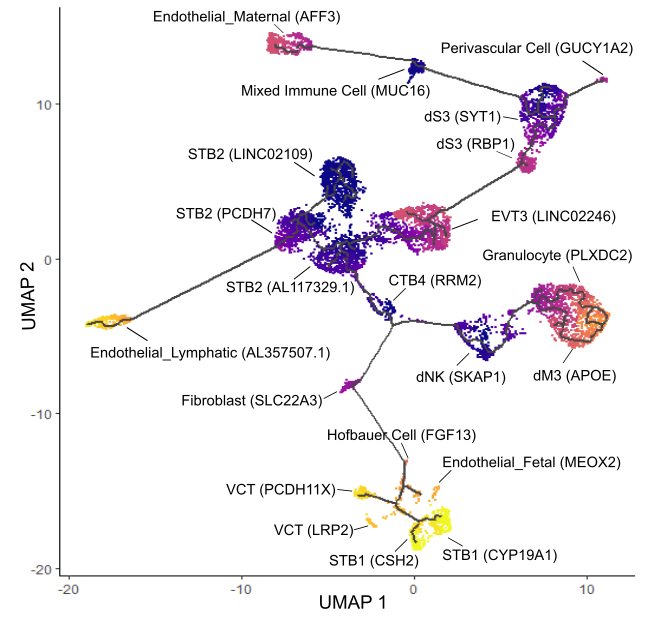

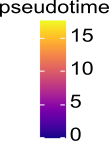

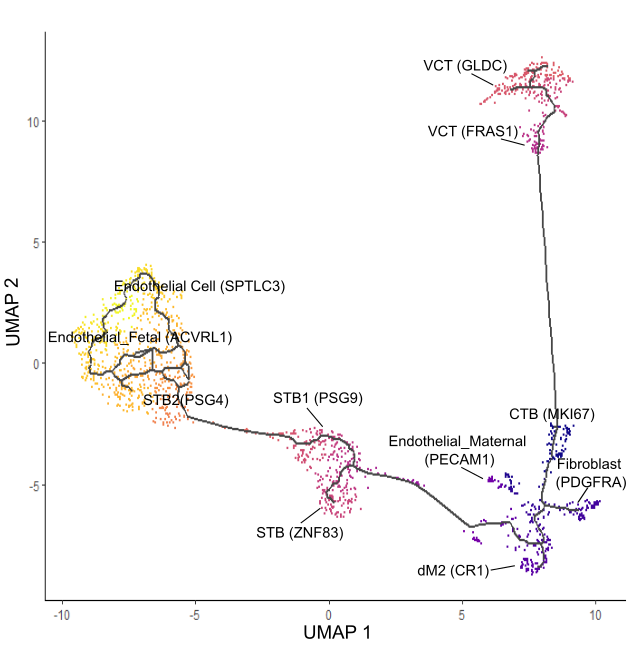

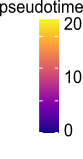


Control

pPROM

sPTL

Figure S3-B


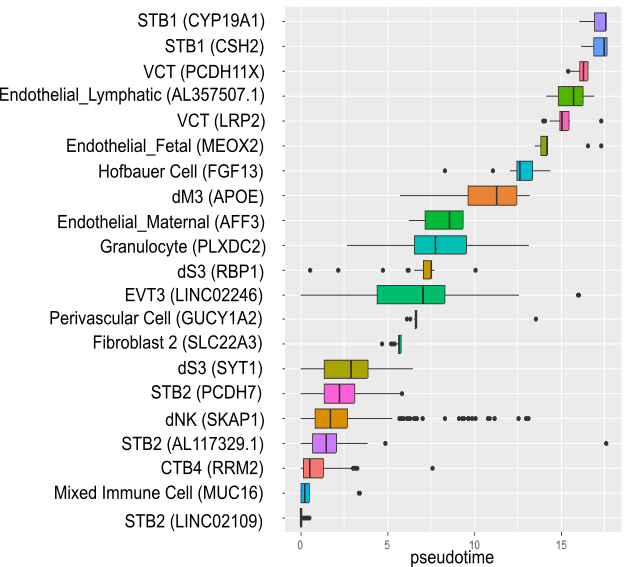

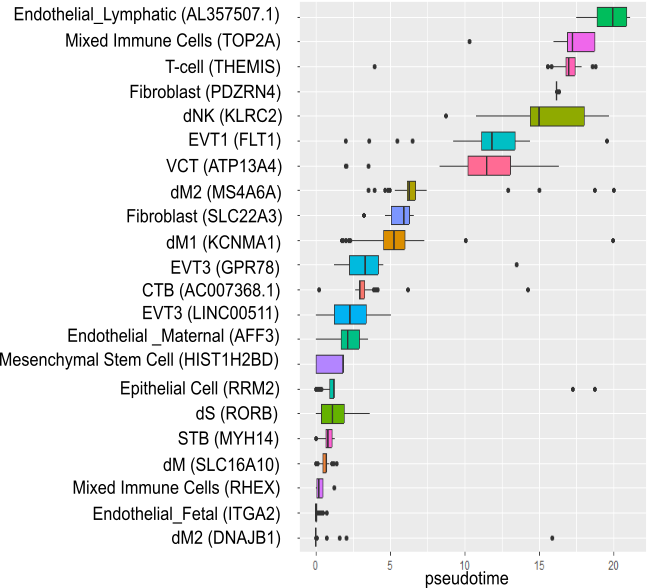

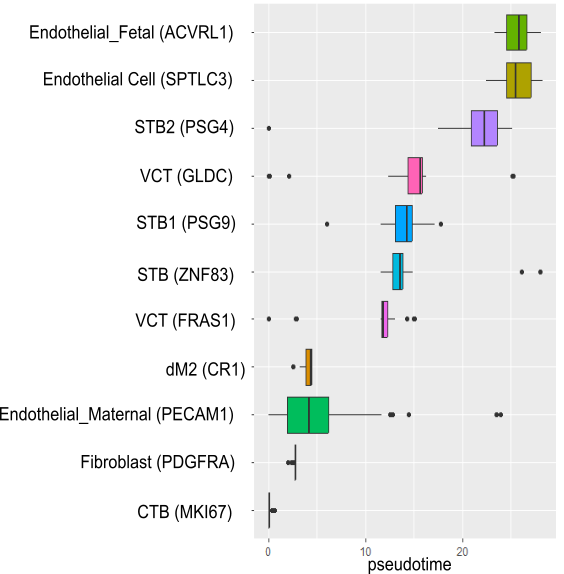


Control

pPROM

sPTL

Figure S3-C


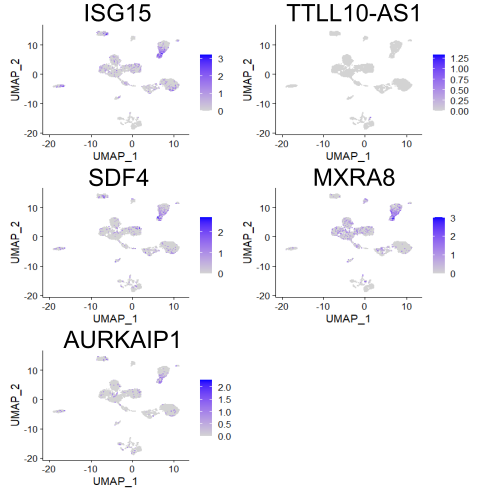

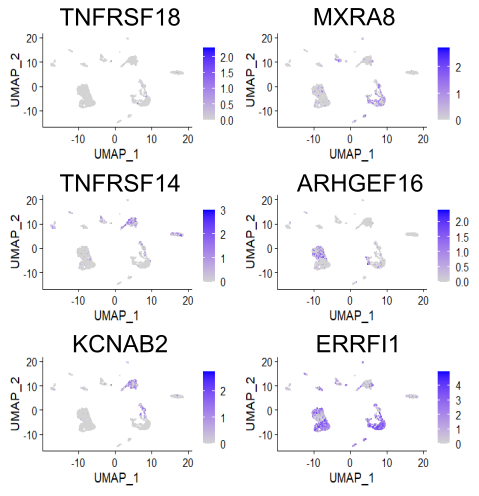

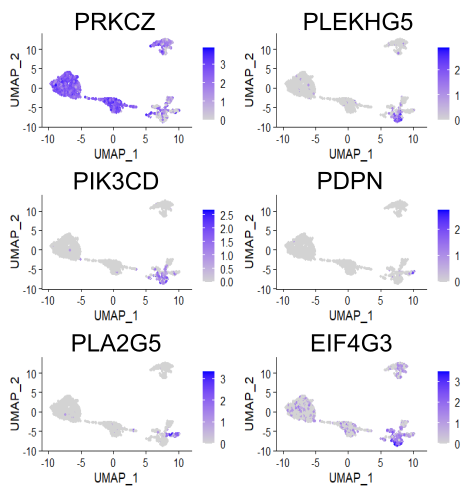


Control

pPROM

sPTL

Figure S4-A


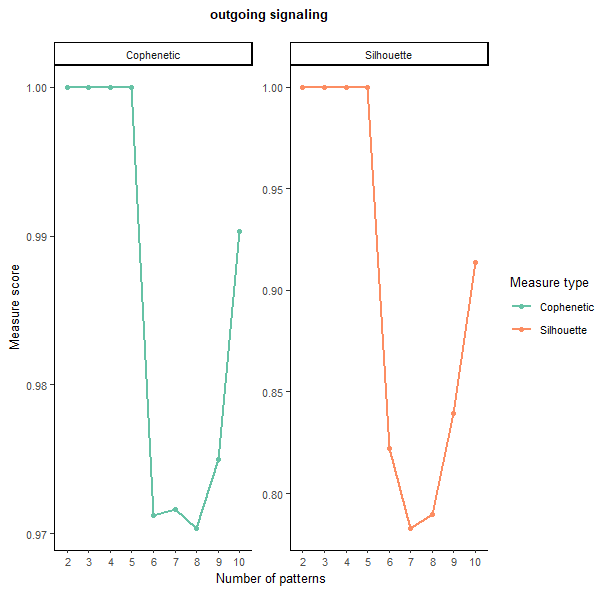

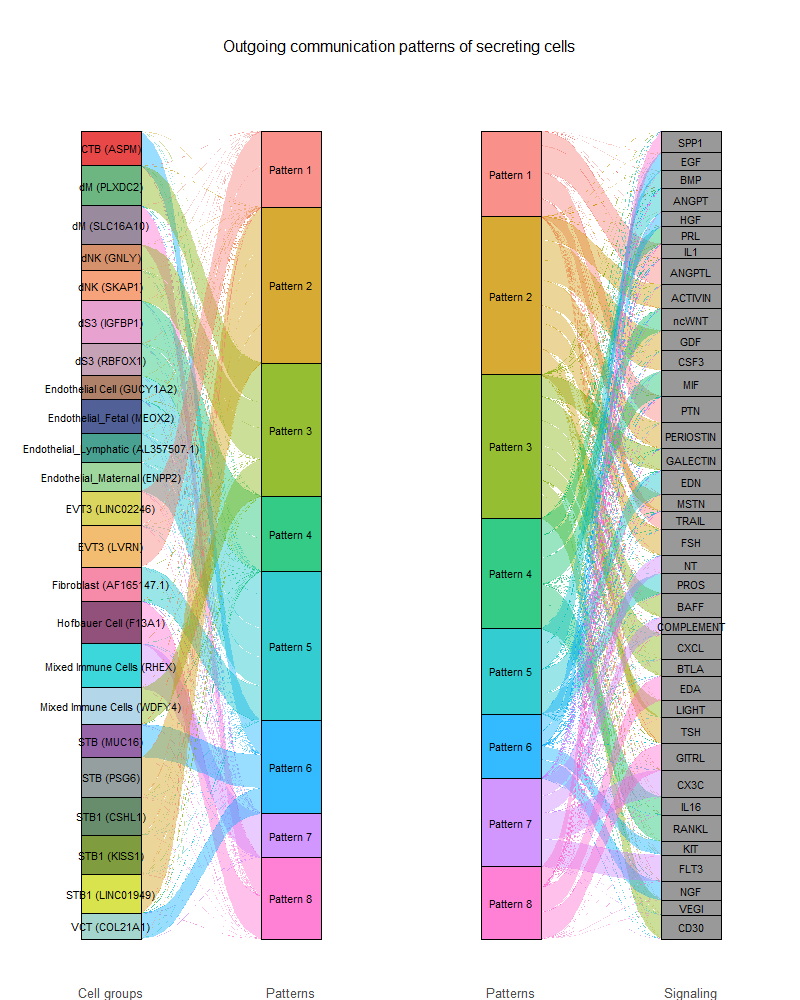


Control Outgoing Signaling


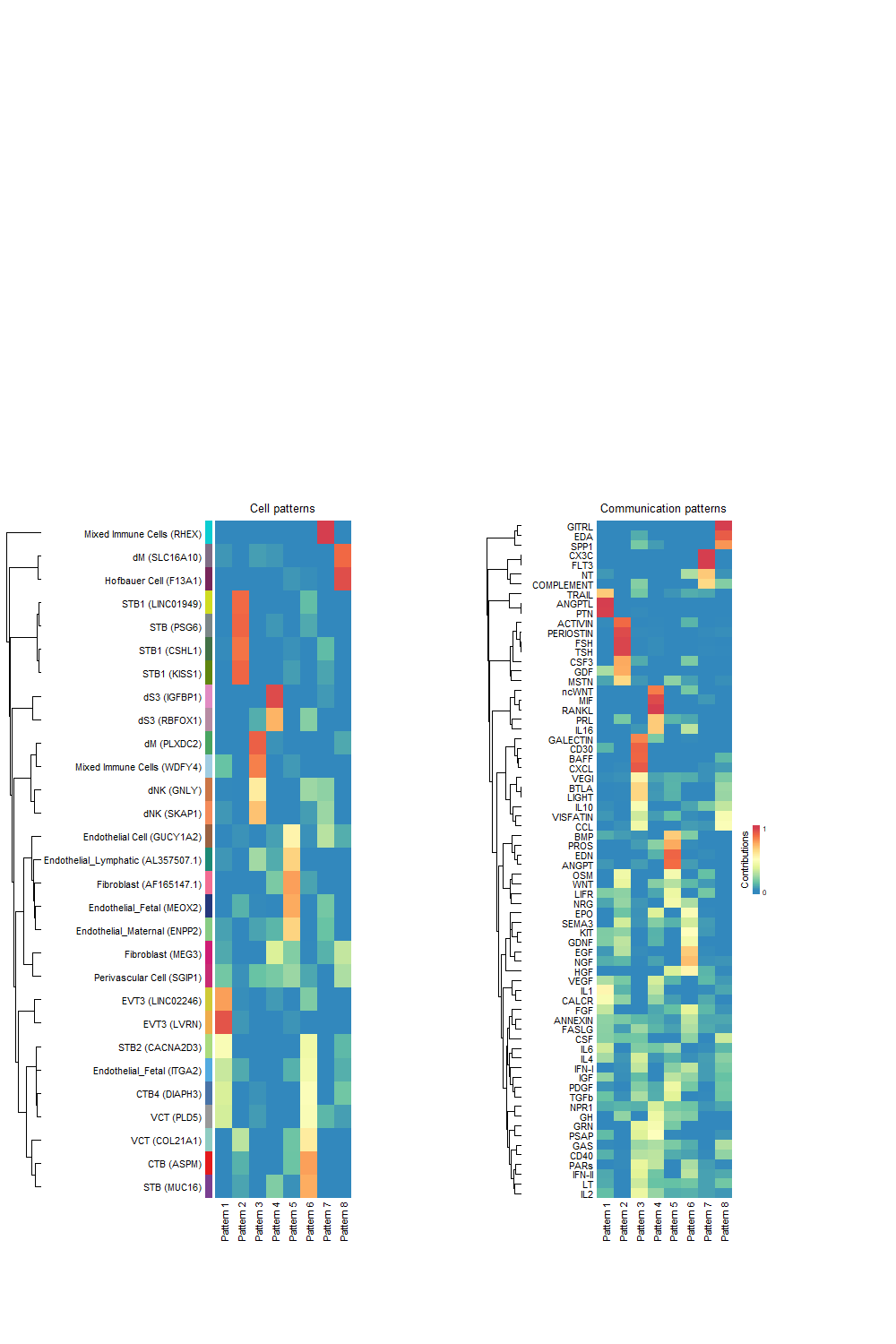


Figure S4-B

pPROM Outgoing Signaling


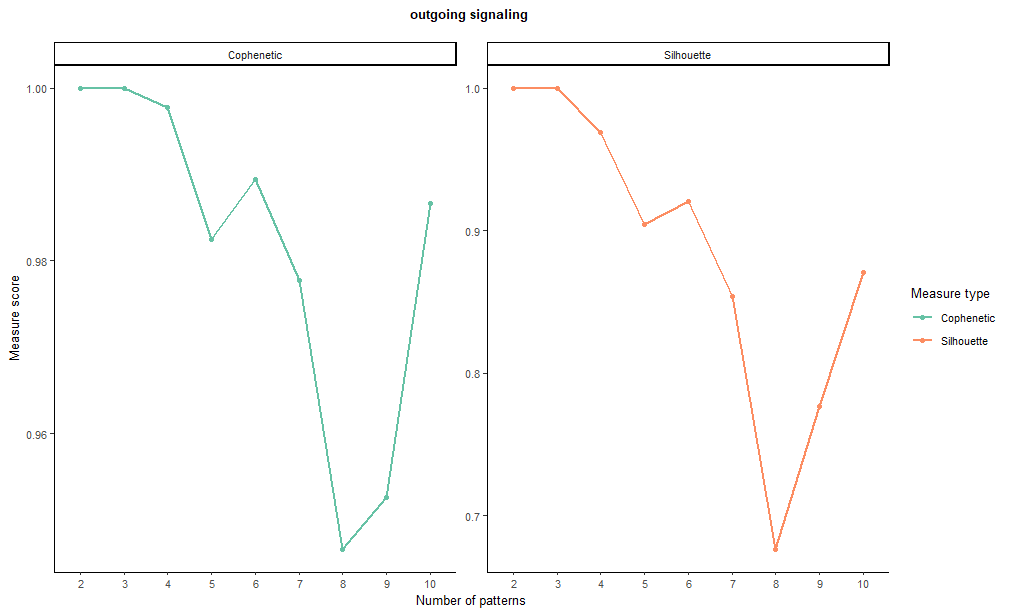

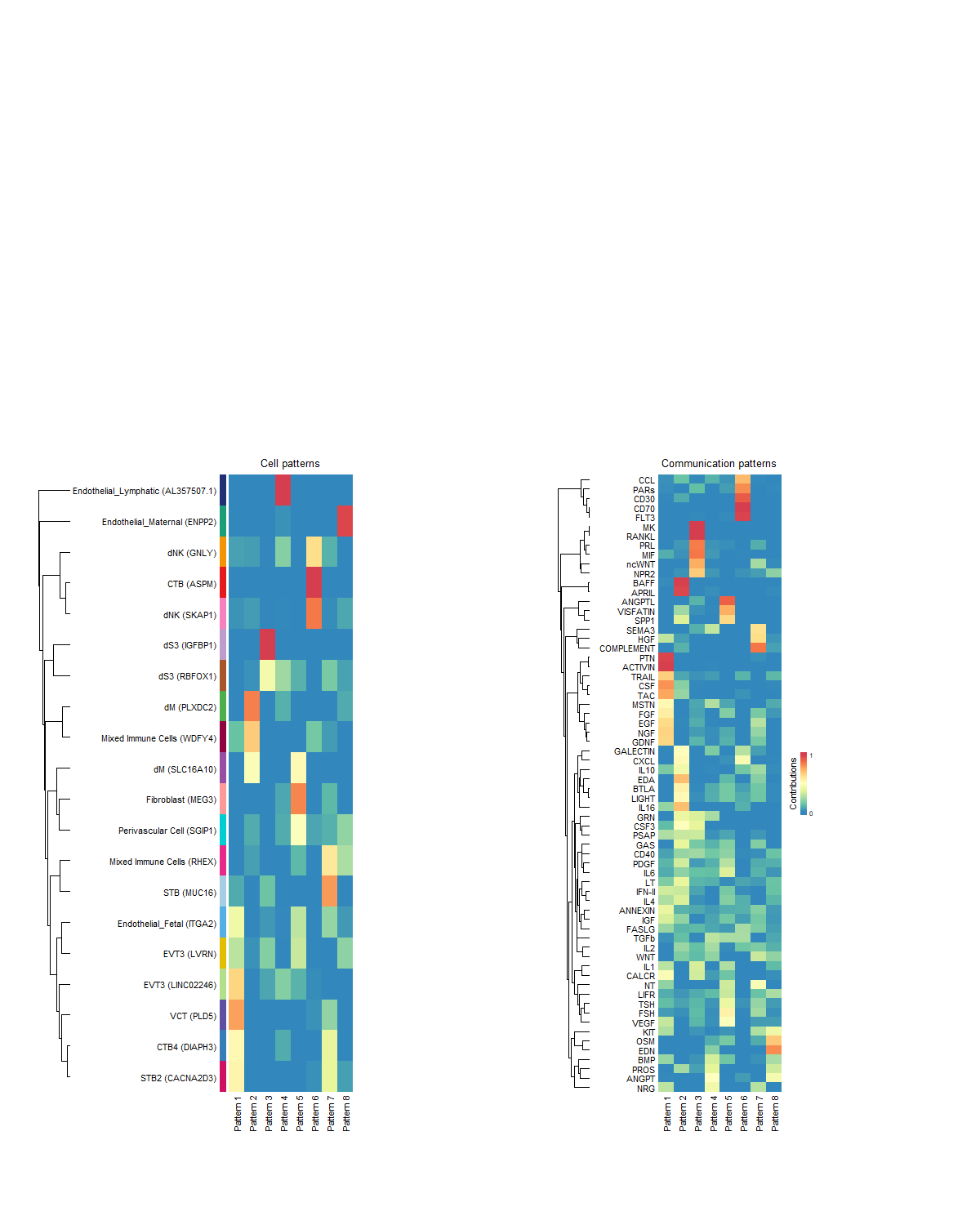

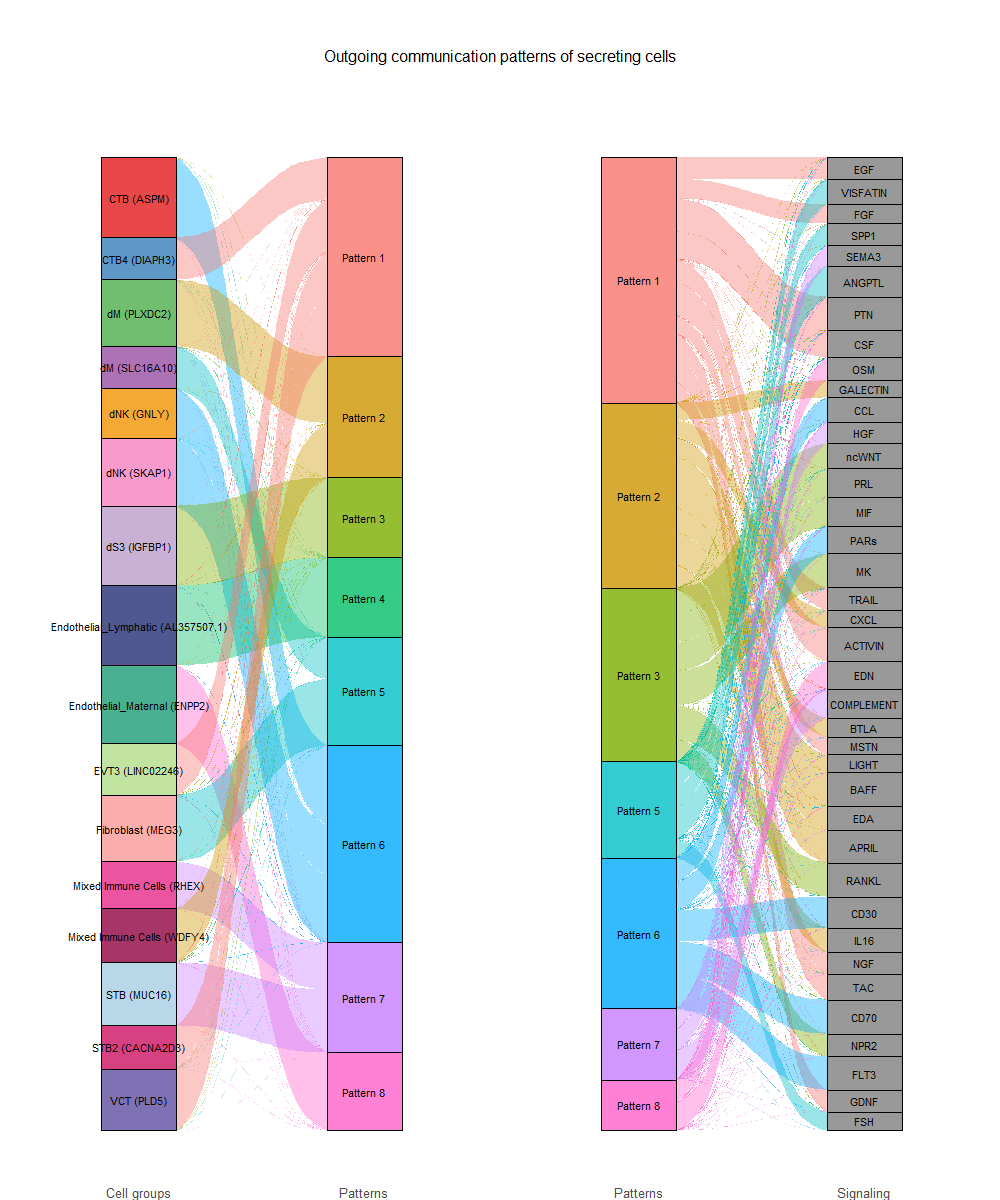


Figure S4-C

sPTL Outgoing Signaling


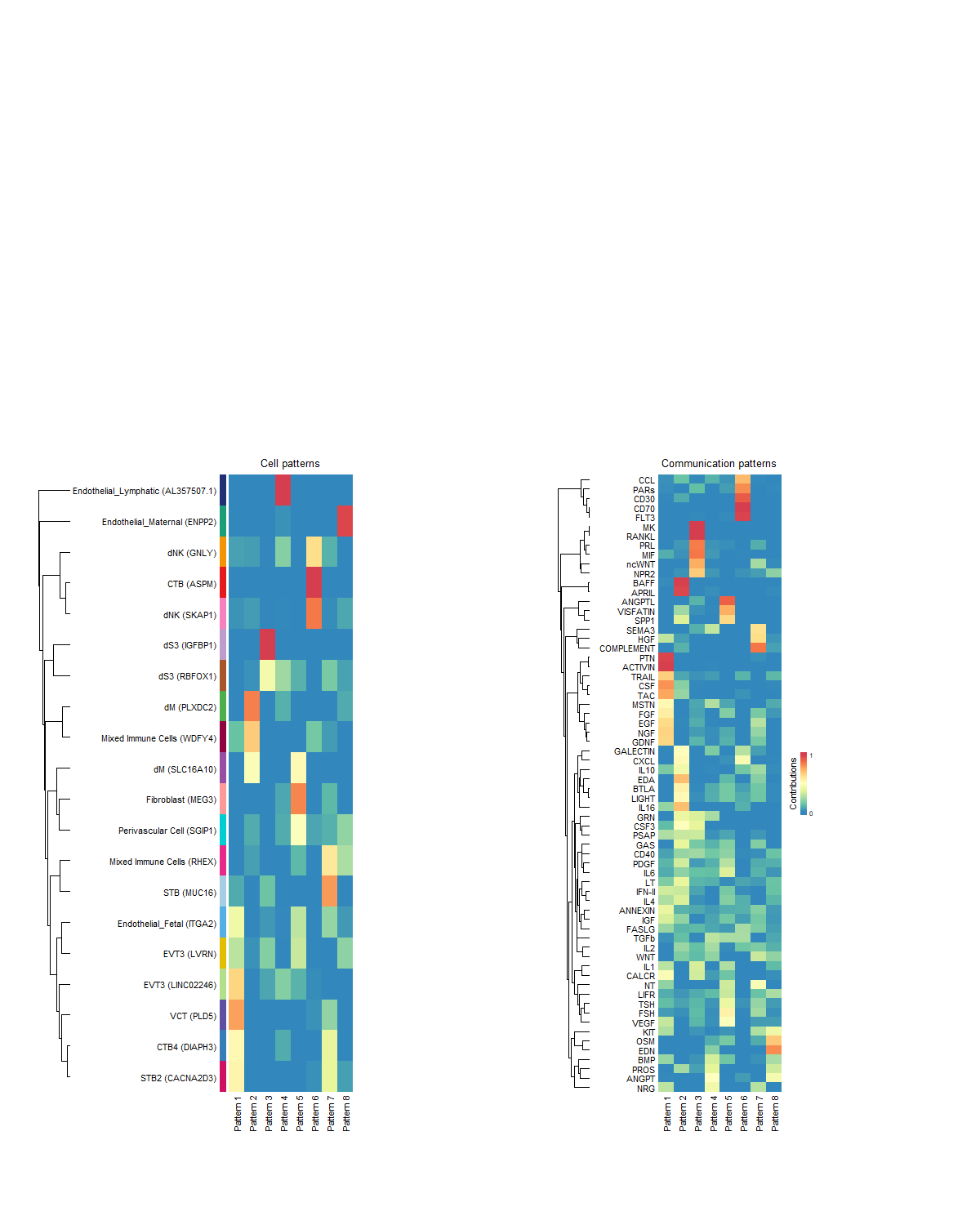


Figure S4-D

Control Incoming Signaling

Figure S4-E

pPROM Incoming Signaling

Figure S4-F

sPTL Incoming Signaling

Figure S5-A

Control

pPROM

sPTL

Figure S5-B

Control

pPROM

sPTL

Figure S6-A

Figure S6-B

pPROM

sPTL
